# Chitosan-based formulations for therapeutic applications. A recent overview

**DOI:** 10.1186/s12929-025-01161-7

**Published:** 2025-07-08

**Authors:** Weronika Gonciarz, Ewa Balcerczak, Marek Brzeziński, Agnieszka Jeleń, Agnieszka J. Pietrzyk-Brzezińska, Vedha Hari B. Narayanan, Magdalena Chmiela

**Affiliations:** 1 Department of Immunology and Infectious Biology, Institute of Microbiology, Biotechnology, and Immunology, Faculty of Biology and Environmental Protection, University of Lodz, Banacha 12/16, 90-237 Lodz, Poland; 2 Department of Pharmaceutical Biochemistry and Molecular Diagnostics, Medical University of Lodz, Muszynskiego 1, 90-151 Lodz, Poland; 3 Laboratory of Molecular Diagnostics, BRaIn Laboratories, Medical University of Lodz, Czechoslowacka 4, 92-216 Lodz, Poland; 4 Centre of Molecular and Macromolecular Studies, Polish Academy of Sciences, Sienkiewicza 112, 90-363 Lodz, Poland; 5 Institute of Molecular and Industrial Biotechnology, Faculty of Biotechnology and Food Sciences, Lodz University of Technology, Stefanowskiego 2/22, 90-537 Lodz, Poland; 6 Pharmaceutical Technology Laboratory, #214, ASK-II, School of Chemical and Biotechnology, SASTRA Deemed University, Thanjavur, 613401 Tamil Nadu India

**Keywords:** Chitosan, Biomedical applications, Nanoparticles, Microparticles

## Abstract

Chitosan is a cationic natural polymer composed of glucosamine and N-acetylglucosamine residues that are held together by a glycosidic bond. Chitosan has many excellent properties, including physicochemical properties, i.e., stability in the natural environment, chelation of metal ions, high sorption properties, biological properties such as biocompatibility and biological activity, ecological properties resulting from biodegradability, and physiological properties, which include non-toxicity, and economic affordability, and is used in various biomedical and industrial applications. The presented article highlights recent developments in chitosan-based formulations for the treatment of bacteria, viruses, cancer, or gastroesophageal reflux disease. Moreover, chitosan-derived biomaterials can also be used in regenerative medicine or food packaging to prevent contamination by pathogenic microorganisms. In summary, this is a valuable compilation in this emerging field that focuses on the biomedical application of chitosan-based biomaterials.

## Background

### The concept of chitosan-based biomedical applications

Chitosan is the most abundant biopolymer with a linear aminopolysaccharide composed of glucosamine and N-acetylglucosamine residues that are held together by glycosidic bonds. It is insoluble in water and soluble in acids such as hydrochloric acid and acetic acid, and its solubility is influenced by the degree of deacetylation. Chitosan is typically extracted from shrimp and crab shells containing abundant calcium carbonate and chitin and can also be obtained from bacteria or fungi [7, 117, 133]. Studies indicate that crustacean shell waste comprises 30%–50% calcium carbonate and 20%–30% chitin by weight, with lobster shells having the highest chitin content of 60%–75% by weight [196]. The extraction process involves demineralization, deproteinization, and deacetylation stages, with demineralization performed first to increase the efficiency of the subsequent steps. The chemical structure of chitosan imparts remarkable biocompatibility and biodegradability [48]. Chitosan can be used for a wide range of applications, including antimicrobial therapies and wound healing, due to its ability to form films and hydrogels, anticoagulant activity and antioxidant activity, and biosorption of heavy metals. Chitosan has been used in various studies to develop carriers to deliver medical formulations, including drugs, plant extracts, microorganisms, and their soluble components. Furthermore, the chelating properties of chitosan make it suitable for wastewater treatment [17]. Under acidic conditions, the protonated amino groups of chitosan confer mucoadhesive properties, facilitating prolonged contact with biological surfaces and promoting drug absorption. Additionally, the versatility of chitosan is evident in its film-forming ability, controlled swelling behavior, and high surface area, which make it suitable for various drug, protein, bacteria, yeast, and microalgae delivery systems, such as nanoparticles Ø 1–1000 nm), microparticles (Ø 1–1000 µm), hydrogels, fibers, and membranes [25, 187, 287]. These formulations can be used externally, topically, or administered parenterally or orally [138, 201].

Chitosan nanoparticles can be produced via the ionic gelation method, exhibiting advantages such as increased stability and high penetration properties. They can also be produced by spray drying. Nanoparticles can be obtained under mild conditions without harmful organic solvents and retain the compound's bioactivity. The stability of chitosan nanoparticles is attributed to the ionic cross-linking of positively charged chitosan with polyanions, which transfer amino groups via protonation. The most commonly used polyanion for ionic cross-linking is tripolyphosphate (TPP), which is nontoxic. The ionic group of TPP interacts with the amine group of chitosan. Zhang et al. (2020) focused on the development characteristics of chitosan-based microspheres obtained via the spray drying technique. They discussed the importance of microspheres in controlled drug delivery because of their high surface area-to-volume ratio and ability to encapsulate hydrophilic and hydrophobic drugs [314]. This study emphasized the influence of various process parameters on the characteristics of the resulting microspheres, highlighting the biocompatibility, mucoadhesive properties, and controlled release capabilities of chitosan [314]. The important feature of chitosan is the ease of chemical modification through primary amino groups at C3 and hydroxyl groups at C3 and C6 in the ring. In drug delivery systems (DDSs), modifications affect the properties of the conjugate, such as its stability, hydrophobicity, pharmacokinetics and pharmacodynamics, solubility, durability, and biocompatibility [138]. However, graft copolymerization and cross-linking, including the formation of polyelectrolyte complexes, are among the reactions leading to extension of the polymer chain and increase in molecular weight; these reactions are also important in chemical methods for obtaining DDSs based on particles [218] and in the design of orally administered pH-sensitive carriers. Mucoadhesiveness is important in designing DDSs and vaccines targeted to mucous membranes [201]. The particle diameters in DDSs are also important in the action of chitosan because microscale particles interact with the mucin layer less easily than nanoscale particles, and it has been shown that particles with diameters less than 200 nm can be internalized into epithelial cells [84].

The mechanism and kinetics of a biological substance’s release from a carrier depend on its physicochemical properties, the polymer (matrix) preparation method, and the particles’ morphology, size, and density. This effect may also depend on the pH and polarity of the dissolution medium in which in vitro release studies are performed [107].

There are three main release mechanisms of cargo, which are preceded by swelling of the polymer due to the inflow of the solvent: (1) diffusion from the surface of the carrier, (2) diffusion through the matrix, and (3) release due to erosion and/or degradation of the polymer. Typically, the release of a substance occurs via a combination of more than one mechanism. In practice, DDSs are designed to achieve the cumulative release of substances within the therapeutic window and with kinetics close to zero order, i.e., regardless of the amount of drug remaining in the system [107]. Binding a certain amount of the active substance on the surface or in the polymer matrix leads to the initial so-called burst effect, the duration of which is directly proportional to the particle diameter [107] and may depend on the encapsulation technique. Using a cross-linking agent can protect against this effect, as can cleaning the particles with an organic solvent, but these methods decrease the loading of the biocomponent into the carrier.

Encapsulation of drugs or biocomponents in the chitosan matrix allows for sustained release, which increases the local drug concentration, and such controlled release of drugs reduces their cytotoxicity. Various materials are used for the encapsulation of biologically active compounds, including nanoparticles/nanocapsules, nanofibers, microspheres, hydrogels [45–47, 221]. Moreover, the nanoparticles based on chitosan can enter the cells’ interior, enhancing the concentration of the drug inside the desired cells. To conclude, in the case of targeted and local drug delivery, it is superior to systematic administration due to long-term and high-dose release of drugs in the desired part of human body, resulting in decreased toxicity to other organs [234].

This review provides an in-depth examination of the research conducted on chitosan and chitosan-based nanoparticles or microparticles. It highlights various drug delivery applications, focusing on antibacterial therapy, particularly for drug-resistant pathogens, anti-Human Immunodeficiency Virus (HIV) therapy, and the ability to use chitosan-like carriers to deliver probiotic bacteria or other bacterial strains with biomedical potential. Moreover, the immunomodulating properties of chitosan are discussed in light of different vaccination strategies and anticancer therapies.

### Biocompatibility, biodegradability and biological properties of chitosan

Owing to its biocompatibility, chitosan has been extensively studied for drug delivery applications with different administration routes, oral, topical, and parenteral, where it facilitates sustained/controlled drug release and prevents drug molecules from decomposing through encapsulation [119]. Chitosan has been characterized as being minimally toxic and is thus generally regarded as safe (GRAS) by regulatory agencies such as the Food and Drug Administration (FDA) when derived from high-quality sources via optimal protocols. Additionally, chitosan displays biocompatible contact properties with living matter and body fluids and exhibits mucoadhesive properties, allowing it to adhere to mucosal gastrointestinal or ophthalmic surfaces to enable the prolonged release of medicaments or ulcers/wound healing. A recent study conducted by Punarvasu and Prashant 2023, demonstrated that low-molecular-weight chitosan can be administered orally for pharmaceutical and food applications [231]. Various experimental approaches have been applied to prove the biocompatibility of chitosan, including cell viability assays, microscopic imaging of cell morphology, and assessments of cell proliferation and cell functions. The 3-(4,5-dimethylthiazol-2-yl)-2,5-diphenyltetrazolium bromide (MTT) reduction assay, lactate dehydrogenase (LDH) release assay, and live/dead staining techniques are commonly used to evaluate cell viability and the extent of damage caused by the application of chitosan and its derivatives [325]. The toxicity of chitosan is dose and time-dependent, and in a wide range of concentrations, chitosan is nontoxic to epithelial cells, fibroblasts, osteoblasts, chondrocytes, and immune cells [135, 182]. Its chemical composition facilitates protein interactions and cell adhesion [249]. This finding was also confirmed in artificial tissue models in which chitosan facilitated cell adhesion, multiplication, and differentiation by providing an appropriate environment for cells and stimulating the extracellular matrix [320]. Since chitosan is mucoadhesive and not hypersensitive, epoetin beta nanoparticles were developed via the ionic gelation technique for topical application in the posterior section of the eye. Chitosan combined with hyaluronic acid showed improved mucoadhesive properties and prolonged drug release [263]. In August 2023, the FDA approved a chitosan product as a primary material for wound healing in humans. A study executed by Matrix Medical Consulting, Inc. (USA) revealed that chitosan promoted a wound-healing effect associated with its antibacterial, noncytotoxic, non-sensitizing, and nonirritant properties [235]. The biocompatibility of chitosan also makes it useful in tissue engineering, where it serves as a scaffold material that supports cell attachment, proliferation, and differentiation—functions essential for the regeneration of various tissues, including skin, bone, and cartilage [24, 109]. Additionally, Rajinikanth et al. explored and documented chitosan's biocompatibility and wound-healing abilities in conjunction with significant antimicrobial properties that directly benefit the wound-healing process [235]. The hemostatic properties of chitosan have been exploited in the development of topical hemostatic agents that can accelerate coagulation and facilitate wound closure, which is crucial in both surgical settings and emergency medicine [160].

Mikušová and Mikuš reviewed advances in chitosan-based nanoparticles for drug delivery [189]. They highlighted the exceptional loading capacity of chitosan nanoparticles, with drug loading efficiencies reaching up to 90%. The unique chemical structure and biocompatibility of chitosan nanoparticles ensure minimal cytotoxicity, with cell viability exceeding 90%, even at high concentrations. The nanoparticles exhibited controlled release kinetics, with sustained drug release profiles lasting more than 72 h, making them promising candidates for targeted and prolonged drug delivery applications. The adaptability of chitosan to chemical modification further broadens its applicability, allowing the development of derivatives with enhanced solubility, strength, and bioactivity tailored to specific medical applications [302].

To confirm the suitability of chitosan-based products from the bench to the bedside, it is necessary to extend cytotoxicity studies for preclinical and clinical practice validation to ensure the safety and efficacy of chitosan-based formulations in vivo to obtain regulatory approval for healthcare application in wound dressings, surgical implants, drug delivery systems, and tissue engineering scaffolds [239].

Furthermore, other important issues to be discussed in terms of usage of the chitosan-based pharmaceuticals are their stability and biodegradability. Chitosan, as a natural polymer, is biodegradable to non-toxic oligosaccharides. Various groups of enzymes are able to degrade chitosan. In human body, these are mainly lysozyme and enzymes of bacteria present in human colon. However, several human chitinases, glucosidases and proteases have been identified as enzymes contributing to chitosan degradation [221]. The overview of the enzymes involved in chitosan degradation was presented by Aranaz et al. [18]. There are numerous factors that influence the stability and biodegradability of chitosan and chitosan-based materials, including molecular weight, polydispersity, deacetylation degree (DD), purity level and moisture content. Among them, the most important ones are molecular weight and DD. Chitosan of higher molecular weight degrades more slowly compared to chitosan of lower molecular weight and in general, the decrease in chitosan molecular weight causes its better adsorption by intestines [126]. DD is the ratio of glucosamine to N-acetylglucosamine units. This parameter usually ranges from 70 to 95% for the commercial chitosan [221]. The highest degradation rate was observed in vitro for DD of 50%, while increasing of DD significantly reduces chitosan degradation and the chitosan of DD above 90% exhibits only minimal degradation. Hence, the chitosan degradation can be controlled by changing the chitosan DD [221]. Additionally, to decrease the possibility of the biodegradation and to improve long-term stability of chitosan-based materials, for instance for storage purposes, various strategies are applied, including the addition of polyols, the preparation of the binary mixtures of chitosan with natural or synthetic polymers (like poly(ethylene oxide) or polyvinylopyrrolidone), and chitosan crosslinking [221]. For instance, the nanoparticles of PEGylated chitosan represent enhanced stability in the bloodstream [287]. Another example, the interactions of chitosan and oppositely charged polyelectrolytes (like sodium tripolyphosphate) enhance the stability of such materials during storage and stress conditions [25]. Similarly, alginate/chitosan microparticles represented increased stability not only in long-term storage but also in simulated gastric and intestinal juices [68]. Thus, the stability and biodegradability of chitosan-based materials can be tunned depending of its intended application.

A structured comparison between native chitosan and its modified forms regarding solubility, stability, and bioactivity is shown in Table 1.

**Table 1:** The comparisons between native chitosan and its modifications regarding solubility, stability and bioactivity

Type of modification	Key achievements	Biomedical applications	Solubility	Stability	Bioactivity	References
Native chitosan	Unknown	Unknown	Soluble in acidic conditions; poor solubility in neutral/alkaline pH	High thermal stability; maintains a crystalline structure	Antimicrobial, antioxidant, haemostatic	[70]
Deacetylated chitosan	Increases solubility, bioactivity	General drug delivery, wound healing	Enhanced solubility in neutral/alkaline pH; solubility increases with higher DD	Stability varies with DD; higher DD can reduce crystallinity, affecting thermal stability	Bioactivity is influenced by DD; higher DD can enhance bioactivity but may increase toxicity	[70, 290]
Depolymerized chitosan	Lowers viscosity, increases solubility	Injectable formulation, nanocarriers	Enhanced solubility in water and acetic acid; solubility increases with decreasing molecular weight	Reduced thermal stability; decomposition temperature decreases with lower molecular weight	Enhanced antimicrobial, antifungal, and antioxidant activities; bioactivity varies with molecular weight	[5, 183]
Quaternization- N-Trimethyl Chitosan (TMC)	High solubility at neutral pH, better stability	Oral/gene drug delivery, tissue repair	Enhanced solubility across a wide pH range due to quaternary ammonium groups	Decreased thermal stability; varies with degree of quaternization, more amorphous structure	Superior antimicrobial, antioxidant, and antifungal activities; pH-independent bioactivity	[79, 116]
Thiolated chitosan	Enhanced mucoadhesion	Mucosal drug delivery	Enhanced Solubility in Neutral and Alkaline Media	Susceptible to oxidation, leading to the formation of disulfide bonds	Mucoadhesion, antioxidant activity, enzyme inhibition, metal ion complexation	[74]
Sulphated chitosan	New bioactivity (neuronal differentiation)	Nerve regeneration	Improves solubility across a wider pH range	Decreases the thermal stability, complex degradation patterns	Anticoagulan, antimicrobia, and anti-inflammatory properties	[48, 188, 223]
Phosphorylated chitosan	Corrosion inhibition	Biomedical coatings	Improved water solubility	Decreases thermal stability and crystallinity	Enhanced bioactivity- antibacterial, antioxidant, and enzyme inhibitory properties	[63, 122, 256]

In the context of health-promoting applications in humans, animals, and plants, the effects of the interactions of chitosan with components of the immune system of living organisms are particularly interesting. The immunomodulatory effects of chitosan are diverse, are dependent on its structure and dosing, and are related to the induction of pro- or anti-inflammatory cytokines. However, the precise definition of the immunomodulatory effect of chitosan is difficult because of the complex character of the inflammatory response, which can be beneficial or deleterious to the host. The inflammatory response is necessary for wound healing, but excessive cellular and humoral inflammatory responses can cause tissue damage. Chitosan has been shown to diminish inflammatory reactions in mice exposed to heat stress, which stimulates oxidative stress in intestinal tissue [195]. This study revealed that, compared with those in the heat stress group not treated with chitosan, the production of heat shock protein (Hsp)-70, toll-like receptor (TLR)-4, protein p65, tumor necrosis factor (TNF)-α and interleukin (IL)-10 in the chitosan-treated group was suppressed on days 1, 7 and 14. In mice inoculated with chitosan, the mRNA levels of the proteins claudin-2 and occludin, which are involved in epithelial cell integrity, were significantly increased.

It has been suggested that the purity of chitosan is essential for its immunological activity. In particular, lipopolysaccharide content, a molecular pattern of infectious agents that activates innate immune cells, may play a role. Ravindranathan et al. analyzed the effects of various biochemical properties (degree of deacetylation, viscosity, polymer length, and endotoxin levels) on the immune responses of antigen-presenting cells (APCs), including macrophages and dendritic cells by assessing the level of TNF-α as a biomarker of cell immunoreactivity [236]. This study revealed that only the endotoxin content and not the degree of deacetylation or viscosity influenced chitosan-induced immune responses. Low-endotoxin chitosan (< 0.01 EU/mg), ranging from 20 to 600 cP and 80% to 97% deacetylation, is inert. However, the structure of chitosan may also be necessary for inducing the activity of immune cells, as shown by Li et al. [162]. Hydroxypropyltrimethyl ammonium chloride chitosan (HACC) and hydroxypropyltrimethyl ammonium chloride fully deacetylated chitosan (De-HACC) were synthesized with various degrees of substitution by varying the ratio of chitosan to glycidyl trimethyl-ammonium chloride (GTMAC). The effects of the degree of quaternary ammonium groups and acetyl groups of these polymers on the immunostimulatory activities of chitosan were examined in RAW 264.7 cells. The levels of nitrogen oxide (NO), IL-6, and TNF-α were compared. The removal of acetyl groups from chitosan improved the degree of substitution of the quaternary ammonium salts, and HACC and De-HACC promoted the activity of immune cells in a substitution-dependent manner: HACC was positively correlated with immune cell activity, and De-HACC was negatively correlated. It was also concluded that the effects of chitosan are driven indirectly by NO, which is upregulated in response to chitosan [160]. A similar conclusion was reached in a study by Chandra and coworkers [41], who investigated the ability of chitosan nanoparticles to induce and augment immune responses in plants and the underlying mechanism. They showed that the treatment of leaves with chitosan nanoparticles significantly improved the plant’s innate immune response through the induction of defense-related genes, including those encoding antioxidant enzymes, and the elevation of total phenolics and NO, which are essential plant signaling molecules.

Knowledge about the signaling pathways induced by chitosan is still insufficient. Shibata et al. explained the macrophage response to chitosan particles in C57BL/6 mice and SCID mice injected intravenously with phagocytose chitin particles [259]. This study revealed that the oxidative burst of alveolar macrophages was increased 50-fold. Furthermore, animals pretreated with monoclonal antibodies against mouse interferon-gamma (IFN-γ) or with natural killer cells NK1.1 presented markedly decreased levels of macrophage activity following the injection of chitin particles. The macrophage priming mechanism induced by chitin particles potentially involves the direct activation of these cells by interferon delivered by NK1.1 CD4- lymphocytes, which may stimulate macrophages via the autocrine pathway [259]. Currently, the best-described intracellular signaling pathways activated in response to chitosan involve GMP–AMP synthase (cGAS), stimulator of interferon genes (STING), and Nod-like receptor (NLR) family pyrin domain containing 3 (NLRP3) [76].

The role of chitosan in the modulation of the immune system has been shown by dietary studies in experimental animal models and farm animals. Li et al. reported that the addition of 500 mg/kg chitosan to the diet affected humoral and cellular immune responses and improved the antioxidative function of beef cattle [162]. The levels of IgM and IgA tended to increase, and total superoxide activity increased, whereas the malondialdehyde content in the serum decreased. A study by Caires et al. on the implantation of tissue-engineered chitosan scaffolds revealed that chitosan interacts with macrophages and increases the secretion of several chemokines, including IL-8, macrophage chemotactic protein-1 (MCP-1), and regulated on activation, normal T cell expressed and secreted (RANTES). Furthermore, macrophages increase stem cell motility within scaffolds by 44% [36].

Interesting results have been obtained from studies on the effects of chitosan on the determinants of immune cell activity in WEHI-3 mice with leukemia [308]. Chitosan increased the total white blood cell number and percentage of CD3-positive T lymphocytes in the animals and decreased the levels of CD19-positive B lymphocytes and CD11b-positive phagocytes after 5 mg/kg treatment and of Mac-3-positive cells after 5 and 20 mg/kg treatment. Moreover, chitosan significantly increased macrophage phagocytosis and the activities of glutamic oxaloacetic transaminase (GOT) and glutamic pyruvic transaminase (GPT) [308].

## The idea of anti-bacterial chitosan formulations

### Direct anti-bacterial activity of chitosan

**Fig. 1:**
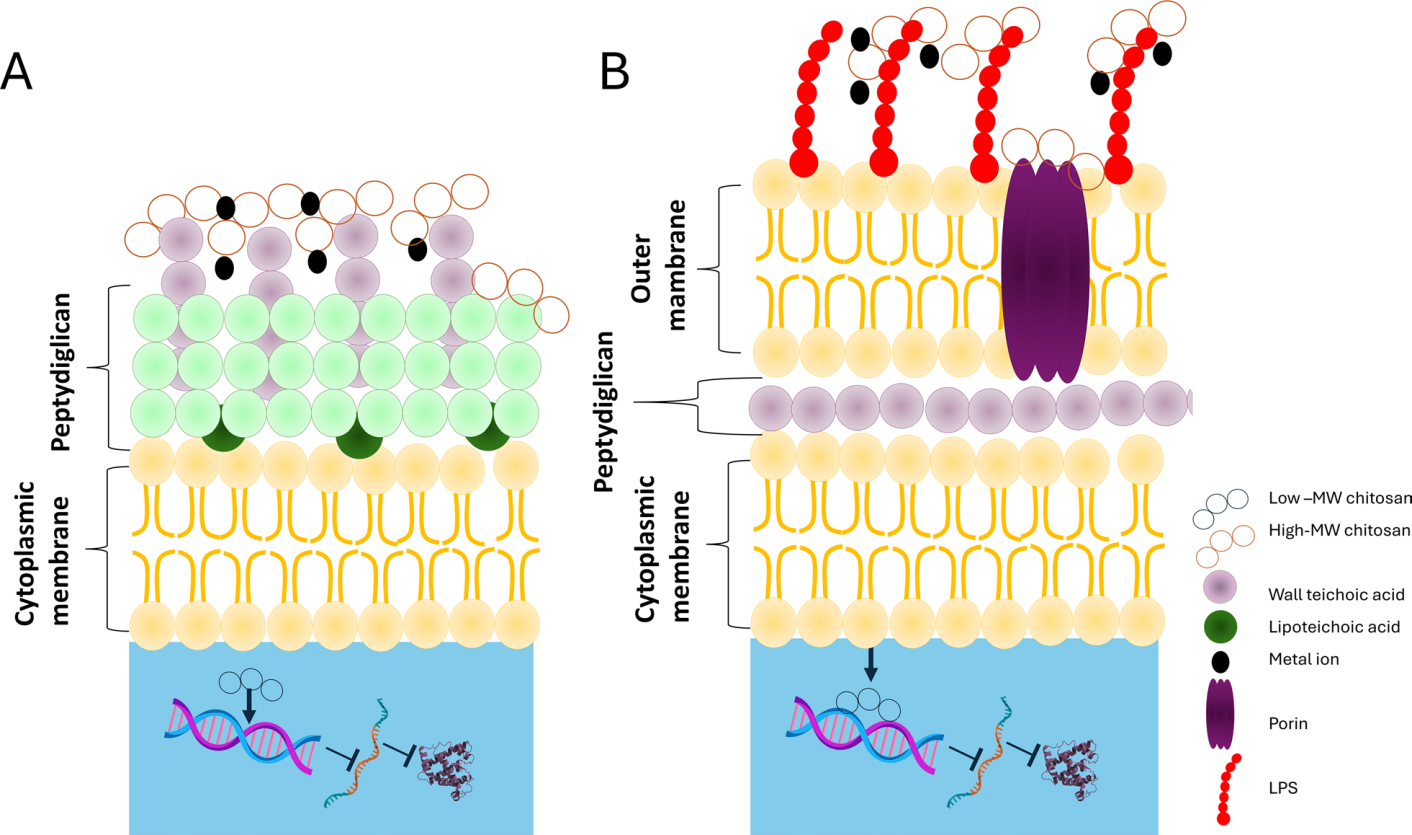
Schematic overview of action modes of chitosan on bacterial pathogens. **A** Gram-positive bacteria, **B** Gram-negative bacteria

Chitosan shows antibacterial and antifungal activity by itself [120, 227, 243, 244, 260]. The mechanism of the bactericidal activity of chitosan depends on its molecular mass, degree of deacetylation, physicochemical properties (concentration, pH, contact time), structure, and reactive hydroxyl groups at the C-3 and C-6 positions. Chitosan may influence the growth of bacterial cells via interactions with bacterial surface structures, metal chelation, or interactions with DNA, as shown in Fig. 1.

The cell wall of Gram-positive bacteria consists of peptidoglycan and teichoic acids (TAs) covalently linked to peptidoglycan. In this group of bacteria, lipoteichoic acids (LTAs) are located in the cell membrane and are negatively charged; therefore, they interact directly with positively charged chitosan. In the cell wall of Gram-negative bacteria, there is a hydrophilic two-dimensional layer containing peptidoglycan, whereas the cytoplasmic membrane is made of lipopolysaccharide (LPS), lipoproteins, and phospholipids; thus, chitosan interacts with anionic components [139]. When the protonated amino groups of chitosan (NH3^+^) encounter an anionic bacterial surface (carboxylic residues, phosphate residues, etc.), the anion moieties may interact electrostatically with the protonated amino groups of chitosan, destroying the bacterial cell barrier and leakage of intracellular substances, especially if the chitosan is low in molecular weight [123]. In this case, chitosan exerts its antimicrobial effect by diminishing the stability of peptidoglycan and changing the osmotic balance of the cell membrane. Chitosan can also compromise the membrane by interfering with electron transport and redox processes [105, 173, 206]. High-molecular-weight chitosan prevents nutrient and oxygen uptake from the intracellular space by creating a polymer film on the surface of the bacterial cell wall. In contrast, low-molecular-weight chitosan penetrates bacterial cells, interacts with DNA, and diminishes protein synthesis, as demonstrated in an *Escherichia coli* (*E. coli*) model [142, 164, 309].

In the pioneer work of Zheng et al., it was concluded that chitosan with high molecular weight (above 166 kDa) possesses a good antimicrobial property against *S. aureus* due to the ability to form a film that suppresses the nutrient adsorptions. In contrast, this effect was inverted for *E. coli*, for which the decrease in chitosan molecular weight (5 to 48.5 kDa) causes the enhancement in its antimicrobial action due to penetration of the bacterial cell wall [323]. Nevertheless, the antimicrobial action against *E. coli* in one investigation is higher for low molecular weight chitosan [75], whereas in other ones, the high molecular weight was most effective [208].

Chitosan exerts a chelating effect, binding essential metals via charged amino groups and thereby inhibiting the growth of microbial agents [147]. This interaction between amino groups and divalent ions within the microorganism cell wall (Ca^2+^ or Mg^2+^) inhibits bacterial growth [102, 194]. These findings suggest that chitosan has better antibacterial activity against Gram-negative bacteria than Gram-positive bacteria because of the presence of LPS, which is often attached to phosphorylated groups, in the cell wall of Gram-negative bacteria [75, 134]. Chitosan binds essential metal ions required for bacterial growth and function. It may occur on bacterial cell surfaces, where it can attach to phosphate groups in lipopolysaccharide (LPS) or other molecules. Such an interaction could cause cell surface instability, potentially rupturing membrane [91]. Chelation occurs through electrostatic attraction facilitated by protonated amine groups. In acidic conditions, the amino groups in chitosan acquire a positive charge due to protonation. These charged amine groups can engage electrostatically with negatively charged regions on the bacterial cell surface, resulting in destabilization of the cell membrane [91, 92]. Chelation and electrostatic attraction can alter the permeability of the bacterial cell membrane, resulting in an osmotic imbalance. This imbalance may cause cell swelling and, ultimately, cell lysis or death. Furthermore, chitosan can disrupt the cell wall by hydrolyzing peptidoglycans, thereby promoting the release of intracellular components [220, 295].

An important question is whether the natural ion-chelating property of chitosan, particularly the ability to bind with divalent cations such as calcium, magnesium and zinc [232, 281], which is beneficial against infection agents will cause adverse effects in vivo as a drug carrier even though the chitosan is considered biocompatible and safe as a drug delivery carrier, particularly for the application of topical, local and temporary use [103, 125]. These characteristics of the chitosan can significantly influence in vivo, especially when used as a drug delivery system. The ions-chitosan complex can modify the drug delivery carrier by localizing to a specific site of action or enhancing loading capacity into the chitosan particulate system [59]. Additionally, the chelation of chitosan with ions improves the carrier system's stability and the drug's release in a controlled manner. However, chelation can also produce adverse effects, such as nutritional depletion from the gastrointestinal tract or bloodstream, which may lead to micronutrient deficiencies. Additionally, the chelation with calcium ions intercellularly could interrupt the cell signaling [268], contraction and functions of muscles and neurons, respectively, which further leads to abnormal processing of the physiological environment [18, 281]. However, most of the studies demonstrated that the chelating effect of chitosan is not strongly evidenced by clinical adverse effects in vivo in both short and long-term use [78].

The degree of chitosan deacetylation, which indicates the percentage of deacylated (glucosamine) units, influences its antibacterial properties and its interaction with bacterial membranes. Higher bacterial cell membranes disruption as a result of chitosan activity may result in an increased production of reactive oxygen species (ROS), which can damage cellular components such as DNA, proteins, and lipids. The effectiveness of ROS-related damage depends on chitosn concentration and duration of cell exposure. The mechanism through which chitosan induces ROS production depends on the disruption by chitosan of electron transportation [309]. Chitosan concentration significantly influences its capacity to inhibit bacterial enzymatic pathways [309]. The pH significantly influences the chitosan charge. At lower pH, chitosan charge is more positive, facilitating its stronger interaction with bacterial cells [190].

Interfering with Quorum Sensing, a communication system in bacteria that is crucial for coordinating virulence factors and the development of bacterial biofilms, can be affected by chitosan due to inhibition of the synthesis of autoinducers (AIs), which bacteria use as chemical signals for communication [251]. It has been shown that chitosan nanoparticles combined with carvacrol effectively reduced violacein production in *Chromobacterium violaceum*, which is regulated by quorum sensing [27]. Chitosan can inhibit AI signaling by interfering with the binding of AIs to their receptors, thereby preventing the activation of the signaling pathways. This can occur through various mechanisms, such as competitive binding with the AI molecule or by altering the receptor's structure [202].

### Chitosan as a matrix for delivery of antimicrobial peptides

Chitosan is an excellent material for delivering antimicrobial peptides (AMPs) and proteins that act as antimicrobial agents. The novel systems used to fight bacteria are listed in Table 2. The presence of primary amino and hydroxyl groups in the chitosan backbone significantly enhances the possible modifications of the prepared materials [226] and, in some cases, facilitates interactions with the AMP or protein to be delivered. Using chitosan-based drug delivery systems allows an increase in the local drug concentration, providing sustained release and simultaneously decreasing systemic toxicity [110]. Additionally, various modifications of the chitosan structure provide stimuli-responsive materials that can direct the drug to infection sites. Furthermore, the encapsulation of AMPs and antimicrobial proteins protects these molecules from proteolytic degradation [33]. Finally, another great advantage of using chitosan in antimicrobial delivery systems is that it also has antimicrobial properties; hence, chitosan has been used as a component of toothpaste [39] and mouthwash [57].

**Table 2:** The list of the described chitosan-based antimicrobial peptide or protein delivery systems

Antimicrobial peptide or protein	Matrix	Materials	Bacterial target	References
Peptides				
Mastoparan	Chitosan	Nanoconstructs	multidrug-resistant (MDR) *Acinetobacter baumannii (A. baumannii)*	[100]
Nisin	Chitosan for coating microcontainers	Microcontainers	pathogenic bacteria colonizing the oral cavity	[29]
Nisin	Carboxymethyl chitosan-nisin nanogels and pullulan as core, and carboxymethyl chitosan/ polyethylene oxide as a shell layer	Electrospun core–shell nanofibers	*Escherichia coli (E. coli), Staphylococcus aureus(S. aureus)*	[67, 293]
Nisin	Chitosan functionalized with DNase I	Nanoparticles	*Listeria monocytogenes (L. monocytogenes)*	[114]
Nisin	Chitosan lactate (CHL) 1:3 ratio with blends of polysaccharides (corn starch, wheat starch, oxidized potato starch, or pullulan)	Films	various food-borne bacteria	[144]
Nisin	Carboxymethyl chitosan	Nanogels	various food-borne bacteria	[294]
Piscidin-1	chitosan crosslinked to β-glycerolphosphate disodium salt pentahydrate	Hydrogels	multidrug-resistant (MDR) *A. baumannii*	[240]
Vancomycin	Chitosan and oleylamine-based zwitterionic lipid-polymer hybrid	Nanovesicles	methicillin-resistant *S. aureus* (MRSA)	[101]
Vancomycin	Chitosan/polyethylene oxide	Electrospun core–shell nanofibers	methicillin-resistant *S. aureus* (MRSA)	[130]
Vancomycin	Chitosan–polylactide	Microspheres	*E. coli, S. aureus*	[154]
Vancomycin	Chitosan-polyaniline	Microgels	*S. aureus*	[160]
Vancomycin	Chitosan photo-crosslinked to pore-closed poly(lactic-co-glycolic acid) microparticles	Hydrogels	pathogenic bacteria colonizing the oral cavity	[267]
Vancomycin	Chitosan and cinnamaldehyde-based thioacetal (CTA) together with ginipin as the crosslinker	Hydrogels	methicillin-resistant *S. aureus* (MRSA)	[285]
GL13K peptide derived from the human salivary parotid secretory protein or innate defense regulator (IDR)-1018 derived from bovine neutrophil host defense peptide bactenecin	Chitosan and pectin derivatives	Nanofiber membranes	pathogenic bacteria colonizing the oral cavity	[30]
Nal-P-113	Chitosan combined with polyethylene oxide	Nanoparticles	pathogenic bacteria colonizing the oral cavity	[115]
Proteins				
Azurin	Chitosan	Nanoparticles	bacterial species related to gastrointestinal cancer biopsies: *Helicobacter pylori (H. pylori), Bacteroides fragilis (B. fragilis), Salmonella enterica (S. enterica), Fusobacterium nucleatum (F. nucleatum),* and *Porphyromonas gingivalis (P. gingivalis)*	[11]
Chimeric endolysin	Chitosan	Nanoparticles	*E. coli, S. aureus, Pseudomonas aeruginosa (P. aeruginosa)*	[1]
Endolysin Cpl-1	Chitosan	Nanoparticles	*S. pneumoniae*	[86]
Endolysin LysMR-5	Alginate-chitosan	Nanoparticles	methicillin-resistant *S. aureus* (MRSA)	[132]
Histatin 3 (HTN3)	Chitosan	Nanoparticles	pathogenic bacteria colonizing the oral cavity	[324]
Lactoferrin	Alginate-chitosan	Microparticles	*Clostridioides difficile (C. difficile)*	[32]

AMPs are short, water-soluble peptides produced by various organisms for host defense. Among the chitosan-based AMP delivery systems, the two most popular are nisin (NIS) and vancomycin (VM) (Table 2). NIS is a bacteriocin produced by *Lactococcus lactis (L. lactis)* and belongs to the lantibiotic family of AMPs, which show antibacterial activity against a broad range of Gram-positive bacteria. Nisin prevents the synthesis of the bacterial cell wall and creates pores in the membrane, causing bacterial cell death. Notably, NIS has GRAS status and is used as a food preservative [65]. VM is a glycopeptide produced by the soil bacterium *Amycolatopsis orientalis* (*A. orientalis*). It also has activity against Gram-positive bacteria and inhibits cell wall synthesis. It is used as an antibiotic for infections caused by bacteria resistant to other antibiotics [200], and the World Health Organization (WHO) classifies it as critically important for human medicine. Most of the discussed chitosan-based materials contain VM or NIS.

#### Chitosan-based AMP delivery systems against multidrug-resistant bacteria

Most recent studies regarding multidrug-resistant bacteria have focused on systems designed to fight MRSA, which is a serious threat to human life and is becoming a global problem [99]. The treatment of skin infections and chronic nonhealing wounds caused by MRSA is especially challenging; hence, some chitosan-based materials have been proposed to deliver VM, one of the antibiotics used in severe cases of MRSA infections [101, 200].

Hassan et al. [101] proposed a formulation of chitosan-based pH-responsive lipid‒polymer hybrid nanovesicles (VM‒OLA‒LPHVs1) carrying VM, where the lipid component was oleylamine-based zwitterionic lipids (OLAs), which also enhance antimicrobial properties. The authors assumed that the resulting polyelectrolyte nanovesicles should have the free amino and hydroxyl groups of chitosan at the surface. In contrast, the carboxylic group of OLA should create electrostatic bonds with VM. In vitro drug release studies revealed that VM release was greater at pH 6.0 than at pH 7.4, indicating that the prepared nanovesicles are pH-responsive. The protonation of the amine groups of chitosan and OLA might cause this. Additionally, acidic pH increased the hydrophilicity of the formulation, which could also result in the breakdown of the system. pH-responsive systems are desirable for the treatment of wound infections, as an acidic pH is present at bacterial infection sites. Further studies revealed that VM-OLA-LPHVs1 were more effective at killing bacteria and eradicating biofilms in a BALB/c mouse skin infection model than free VM [101].

Another type of material proposed for wound healing is a system of hybrid chitosan/polyethylene oxide (PEO) nanofibers loaded with VM. PEO, similar to chitosan, is biocompatible. It can form hydrogen bonds with chitosan, leading to chain stiffness of the resulting nanofibers. The concentration of VM in the nanofibers was 2.5 or 5% (w/v), and in vivo studies revealed that a lower antibiotic concentration was optimal. The wound area of the 2.5% VM group of rats was smaller than that of the 5% VM group, indicating a more efficient healing process [130].

The VM-bearing hydrogel was prepared by grafting chitosan and cinnamaldehyde-based thioacetal (CTA) with ginipin as the crosslinker. The covalently cross-linked chitosan hydrogels can adsorb large amounts of liquid. The inclusion of CTA in the formulation was advantageous because of two properties [285]. First, CTA was proven to sense ROS [301] thus, the produced material became stimuli-responsive [287]. Second, exposure to ROS causes the release of cinnamaldehyde from CTA, and cinnemaldehyde has bactericidal properties, as it destroys the bacterial cell wall. The hydrogel was tested in vivo in a mouse full-layer skin defect model. It accelerated wound healing and skin regeneration processes and contributed to improved VM bioavailability, which makes it an excellent material for the treatment of MRSA-induced skin infections [287].

Chitosan-based AMP delivery systems for the treatment of *S. aureus* infections are not limited to wound healing. VM-chitosan-polyaniline microgels [156] and VM-chitosan-polylactide microspheres [160] have been proposed for the delivery of VM to the inflamed intestine or to infected bone tissue, respectively. The microspheres were prepared using various ratios of amino Schiff base chitooligosaccharides to lactide, and the most efficient drug release was obtained at a ratio of 1:00. The microspheres exhibited antibacterial properties against *S. aureus* and *E. coli* [154]. VM-chitosan-polyaniline microgels were designed for lysozyme-triggered VM release. Lysozyme cleaves the glycosidic bonds of chitosan, which results in drug release. Interestingly, intestinal pathogens lead to cell dysfunction, and consequently, lysozyme is secreted in more significant amounts than in healthy cells. Hence, the proposed microgels can be used to treat intestinal infectious diseases, such as inflammatory bowel disease, and can target only the infected sites of the intestine without harming healthy tissue. The tests performed in the simulated inflammatory intestinal microenvironment confirmed this behavior of the microgels. Additionally, VM-chitosan-polyaniline microgels are stable at the acidic pH values in the stomach [165].

Another dangerous multidrug-resistant bacterium that can infect wounds is *A. baumannii. *In vitro*,* studies of AMP-loaded thermoresponsive chitosan hydrogels confirmed that this material is cytocompatible, as tested on Hu02 fibroblasts. These cells exhibited appropriate attachment and growth on the hydrogel. Thermoresponsiveness was achieved by the use of β-glycerolphosphate disodium salt pentahydrate as a cross-linker. The AMP loaded into the chitosan hydrogel was piscidin-1 [240], a peptide originating from aquaculture fishes. Piscidin-1 has a broad range of antimicrobial activity against Gram-positive and Gram-negative bacteria, yeast, and fungi; however, its cytotoxicity to red blood cells limits its use [148]. Hence, its utilization in a hydrogel that can be applied as a topical antimicrobial agent reduces its toxic effects [240]. *A. baumannii* not only infects wounds but also causes pneumonia, urinary tract infections, and septicemia. Thus, it is also called a superbug, and along with *S. aureus*, it has been included in the top six most dangerous pathogens (ESKAPE) [291]. Chitosan-based nanoconstructs loaded with mastoparan, another AMP, were prepared to fight these bacteria [100]. Mastoparan is a peptide extracted from wasp venom that disrupts the bacterial cell membrane, leading to increased permeability and cell death [112]. The preparation of the nanocomplex was preceded by molecular dynamics simulations, which demonstrated that chitosan cross-linked with sodium tripolyphosphate and mixed with mastoparan most likely forms circular rings encasing the mastoparan. The nanoconstructs caused bacterial damage during in vitro studies and reduced the number of bacterial colonies in BALB/c mice (sepsis model) [100].

#### Chitosan-based AMP-delivery systems against oral cavity bacterial pathogens

Various chitosan-based materials have been designed to deliver AMPs to infection sites in the oral cavity. Many microbes that colonize the oral cavity have beneficial effects on human health. However, they sometimes form biofilms of pathogenic bacteria that cause periodontitis and dental caries [60]. An AMP-delivery system targeting multispecies bacterial biofilms was proposed. It is based on miniaturized devices, called microcontainers (MCs), loaded with nisin. MCs were functionalized with a lid made of chitosan, taking advantage of its mucoadhesive properties. These bioadhesive MCs enable the retention of nisin in the oral cavity. These MCs are not easily removed by flowing saliva and are more effective than free nisin [29].

Another system that is dedicated to fighting periodontitis, especially periodontitis related to root caries, is based on PEO combined with chitosan nanoparticles loaded with a novel AMP, Nal-P-113 [115]. This peptide is a modification of peptide P-113, which is currently used in various products available on the market. The histidine residues of P-113 are replaced in Nal-P-113 with β-naphthylalanines, which increases the ability of the peptide to penetrate deeper into the bacterial cell membrane [283]. In vitro studies revealed that the proposed nanoparticles inhibited the growth of *F. nucleatum*, *Streptococcus gordonii,* and *P. gingivalis*. In addition, they are efficient at inhibiting bacterial biofilms [115].

The bioadhesive properties of chitosan have also been exploited to develop nanofiber membranes that adhere to both soft mucosal and hard bone/enamel tissue. The dual nature of the material was achieved by coating chitosan membranes with oxidized pectin. The resulting nanofiber membranes exhibit moderate and reversible underwater adhesion properties. Additionally, the membranes are pH-responsive, which is a desired feature of such materials, as the oral pH is approximately 6.7, whereas it decreases as far as 4.5 during infection. The nanofiber membranes were loaded with GL13K peptide, derived from the human salivary parotid secretory protein, and innate defense regulator (IDR)-1018, derived from the bovine neutrophil host defense peptide bactenecin. Both peptides have antimicrobial properties and were released in a pH-controlled manner in the in vitro studies [30].

Song et al. proposed a chitosan-based hydrogel that delivers not only the AMP vancomycin but also recombinant human bone morphogenetic protein-2 (rhBMP-2), which promotes osteogenesis [267]. Hence, hydrogels have great potential in supporting the dental implantation process. The proposed hydrogel combined chitosan and pore-closed poly(lactic-co-glycolic acid) microparticles. The chitosan matrix was loaded with VM, whereas the microparticles contained rhBMP-2. This formulation enabled the sequential release of the peptide and the protein, as shown by in vitro and in vivo studies. VM was released rapidly for the initial two days, while rhBMP-2 was released in a sustained manner for approximately 12 days. Thus, the hydrogel protected the implantation site from infection and promoted osteointegration of the dental implant [267].

#### Chitosan-based AMP delivery systems as food packaging materials prevent the development of pathogenic microorganisms

The antimicrobial properties of peptides have also been utilized to prevent the growth of various foodborne bacteria and improve the safety of stored food products. Chitosan, a biodegradable polymer, is used as a matrix to prepare different materials dedicated to food packaging and carrying AMPs. In all the examples described here, the AMP of choice was NIS [111, 144, 294].

Kowalczyk et al. performed comparative studies of films based on blends of chitosan lactate and one of the following polysaccharides: corn starch, wheat starch, oxidized potato starch, or pullulan [144]. The incorporation of NIS into the films at acidic pH protected NIS from degradation, as it is inactive under alkaline conditions. The blends were prepared using a 75:25 polysaccharide: chitosan lactate ratio. Various concentrations of NIS were introduced into the mixture prior to casting the material on trays and drying, which led to the formation of films. Pullulan offers antifungal activity, which is also desirable in packaging materials; hence, this polysaccharide was also tested. The NIS release kinetics were studied in water, and the obtained films were water soluble; thus, at the final stages of the experiment, NIS was wholly released from the films. NIS was released more slowly from starch/chitosan lactate films than from pullulan-containing films, which was preferable, as AMP should be released slowly during food storage. All formulations exhibited similar antimicrobial activity and limited the growth of *Bacillus cereus (B. cereus), L. monocytogenes, S. aureus*, and the phytopathogen *Pectobacterium carotovorum* (*P. carotovorum*) but did not inhibit the growth of *E. coli* or *S. enterica* ssp. *enteretica* sv. Anatum [144].

Chitosan and pullulan have also been combined to prepare electrospun core–shell nanofibers dedicated to fish storage and protection from spoilage. The nanofibers were prepared via coaxial electrospinning. Pullulan was the core of the fibers, whereas carboxymethyl chitosan (CMCS)/PEO constituted a shell layer. Moreover, CMCS-NIS nanogels were obtained via self-assembly and loaded into the core of the nanofibers. This material exhibited good thermostability, mechanical strength, and antibacterial properties when tested against *S. aureus* and *E. coli.* Additionally, it was used for the storage of bass and extended the shelf life from 9 to 15 days [63]. Another CMCS-based material for food packaging was a nanogel loaded with NIS and prepared via a combination of electrostatic self-assembly and chemical crosslinking. Like the described nanofibers, the nanogel exhibited antibacterial activity against *S. aureus* and *E. coli* [294].

Chitosan nanoparticles loaded with NIS were prepared to inhibit the growth of *L. monocytogenes*, a food-contaminating bacterial species that causes life-threatening infections and economic losses. The nanoparticles were further functionalized with DNase I by covalent grafting of the enzyme onto the nanoparticle surface [114]. DNase I degrades eDNA, which is essential for biofilms' structural stability and promotes bacteria's aggregation and intercellular adhesion [215]. Hence, the use of this enzyme in the formulation enhanced the reduction in *L. monocytogenes* biofilm formation on polyurethane [114].

### Chitosan-based antibacterial protein delivery systems

The use of proteins as antimicrobial components of drug delivery systems is even more complicated than the use of peptides. Proteins are much larger and much more fragile and prone to proteolytic degradation, and many factors can cause their denaturation. The loading of proteins into chitosan-based nano- or microparticles significantly extends the in vivo half-life of the proteins. Hence, there have been trials describing the utilization of several proteins in chitosan-based drug delivery systems aimed at fighting bacteria (Table 2).

Among proteins with antibacterial properties, endolysins represent a large group of novel antimicrobial agents. These proteins are also classified as enzybiotics, as their antibacterial properties are closely related to their enzymatic activity. Endolysins are peptidoglycan hydrolases originating from bacteriophages, and according to their class, they target peptidoglycans of the bacterial cell wall. They are effective against life-threatening bacteria such as MRSA, as bacteria currently exhibit low resistance to these molecules. These enzymes usually comprise two domains: an N-terminal catalytic domain and a C-terminal domain responsible for binding to the bacterial cell wall [94]. The recombinant proteins provide the possibility to modify the protein of interest further, and such an attempt was made to produce a chimeric endolysin composed of the N-terminal domain representing cysteine/histidine-dependent amidohydrolase/peptidase (CHAP) and the C-terminal domain originating from the endolysin LysK amidase-2 domain connected by a decapeptide linker. The chimeric protein reduced the growth of MRSA [95] and was thus chosen to prepare nanoparticles against different bacteria. Two types of chitosan nanoparticles were designed: in one of the proposed formulations, chimeric endolysin was attached covalently to the nanoparticles, whereas in the other formulation, the chimeric protein was noncovalently entrapped in the nanoparticles. The lytic activity of the nanoparticles was shown against *S. aureus, E. coli*, and *P. aeruginosa.* Furthermore, a synergistic effect between the nanoparticles and VM was observed. Additionally, the nanoparticles effectively reduced biofilm formation by *E. coli* [1].

Cpl-1 is another endolysin used for the preparation of nanoparticles. This drug delivery system was developed to fight antibiotic-resistant *S. pneumoniae*. The mucoadhesive properties of the chitosan nanoparticles were tested ex vivo*,* and the results confirmed the mucoadhesive nature of the formulation. Moreover, in vitro and in vivo studies have shown that these nanoparticles are biocompatible, noncytotoxic, and able to stimulate the immune system of tested mice [86]. Both chimeric endolysin and Cpl-1 were entrapped in chitosan nanoparticles prepared via ionic gelation with the addition of sodium tripolyphosphate (TPP) [1, 86]. In contrast, the preparation of the delivery system for endolysin LysMR-5 involved the use of sodium alginate. The process started with the mixing of sodium alginate with LysMR-5. Then, the pregelation of the alginate core loaded with LysMR-5 was induced by calcium ions, and the next step was complexation with chitosan. The gelation method for the production of nanoparticles requires mild conditions that are safe for proteins. The proposed LysMR-5-delivery system was tested in vitro*,* and it exhibited antibacterial properties against *S. aureus* and was not cytotoxic [132].

Proteins encapsulated in chitosan nanoparticles can also be used to prevent dental caries. Histatins (HTNs) are salivary proteins that act in the oral cavity to maintain homeostasis and exhibit antibacterial properties. HTN3, a representative histatin, was used to prepare nanoparticles that target bacteria in the oral cavity. Here again, the ionic gelation method with TPP was utilized. Interestingly, in vitro studies revealed that the chitosan nanoparticles with and without HTN3 both showed antibacterial properties against *S. mutans*, a bacterium that plays a significant role in forming dental caries, and reduced biofilm formation. Chitosan also has antibacterial properties; in this case, the nanoparticles efficiently killed the bacteria even when HTN3 was absent [324]. In other reports, the release of AMP or proteins with antimicrobial properties usually significantly improved the antibacterial effect of the material. It is possible that more differences could be observed when the tests include additional bacterial species.

Some proteins have antibacterial and anticancer properties. An example of such a protein is azurin, which is produced by the pathogenic bacteria *P. aeruginosa*. Notably, in some cancers, such as gastrointestinal cancer, various bacteria are detected in biopsies from patients. These bacteria include *H. pylori, B. fragilis, S. enterica, F. nucleatum,* and *P. gingivalis,* which can all contribute to cancer development [108]. Azurin immobilized via adsorption on the surface of chitosan nanoparticles was shown to exhibit antibacterial and anticancer properties [11]. A different approach was used to design a delivery system for bovine lactoferrin, a protein that exhibits antibacterial properties against *C. difficile*, a dangerous pathogen of the colon. The authors of this study aimed to deliver the protein to the colon via the gastrointestinal tract. Thus, factors such as changing pH values in different parts of the tract are essential. Bovine lactoferrin was encapsulated in alginate microparticles via gelation or emulsification methods. The microparticles were coated with chitosan. The release of the protein was tested at different pH values that simulate various conditions in the gastrointestinal tract. No release was noted at acidic pH values, whereas pH 7.4, which mimics the colon environment, caused the release of most of the encapsulated lactoferrin. The microparticles were also applied to human intestinal epithelial cells and reduced the cytotoxic effects of *C. difficile* toxins A and B [32].

**Fig. 2:**
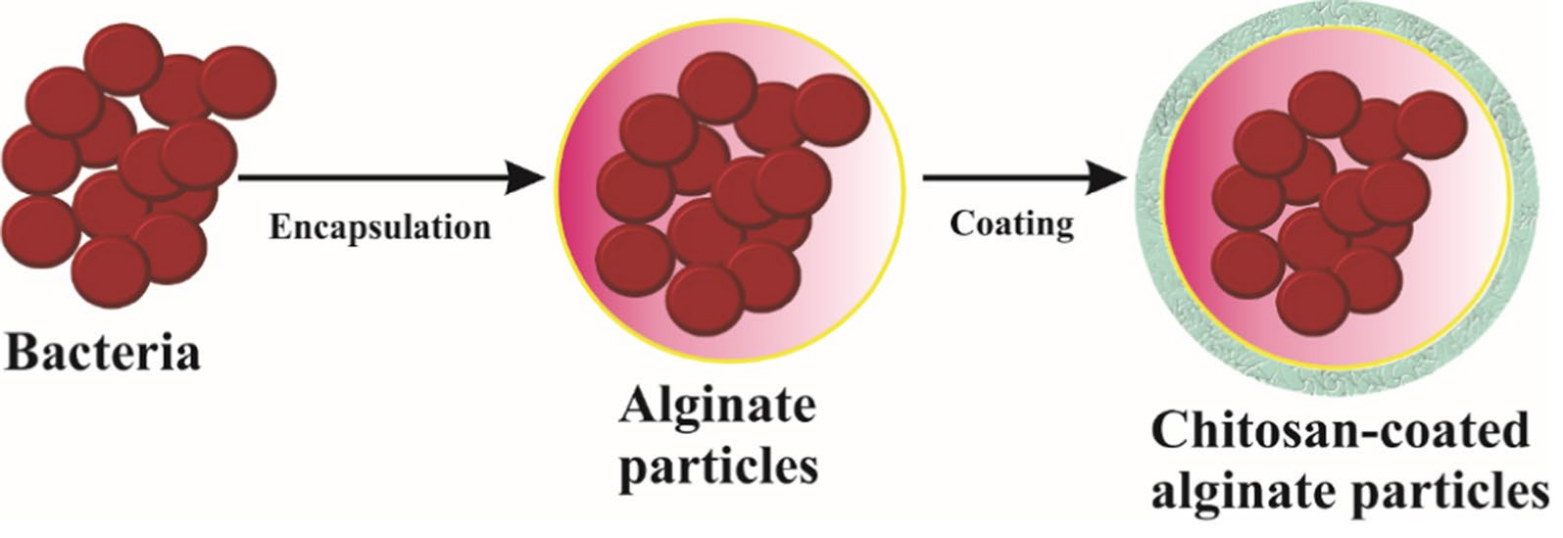
Scheme of most common encapsulation of bacteria in particles composed of alginate core and chitosan shell

In summary, proteins can be successfully encapsulated in chitosan nano- or microparticles or immobilized on chitosan materials. The size of the proteins used in the studies varied from 50 (for HTN3) and 128 (for azurin) to 689 amino acid residues (for bovine lactoferrin). A comparison of the available spatial structures of azurin and lactoferrin (Fig. 2) revealed that the dimensions of such molecules are less than 10 nm (100 Å); hence, it is possible to pack a certain amount of such molecules in nano- or microparticles. The mild conditions used to prepare these materials enable the proper function of the proteins at the target sites.

The chitosan showed immunogenic properties when used as a drug delivery carrier for proteins or as adjuvant in vaccine formulations. This activity was found to be dose-dependent and influenced by the chitosan structure [14, 80]. In the study by Koppolu and Zaharoff [143], the bovine serum albumin labelled with fluorescein isothiocyanate (FITC-BSA) or ovalbumin (OVA) were loaded in particles developed by the precipitation-coacervation method. The effectiveness of encapsulation reached 89% and particle size was in the range 3–300 µm. These particles were engulfed by antigen presenting cells (APCs), which were then activated. Another study showed that chitosan was involved in modifying the maturation, activation, cytokine production, and polarization of dendritic cells (DCs) and macrophages both involved in the development of innate and adaptive immune responses, and the potential mechanisms are based on modulation of different signaling pathways, including cGAS-STING, STAT-1, and MLRP3 [80]. Oliveira et al. showed [211] that in response to chitosan particles, the activity and motility of macrophages were increased. The study by Scherlie et al., [252] revealed that chitosan-driven immunomodulatory effects were associated with different physicochemical characteristics of chitosan, including molecular weight, particle size, extraction technique, and degree of deacetylation (DDA). A lower degree of DDA (76%) resulted in higher reactivity of immune cells compared to chitosan with higher DDA (81%). Low chitosan dose induced an anti-inflammatory response related to releasing IL-1ra without activation of inflammasomes while a high dose of chitosan caused disruption of lysosomes present in immune cells resulting with an activation of inflammasomes and pro-inflammatory response [76]. In has been shown that chitosan induces type-I interferon response driven by activation of cGAS-STING signaling pathway [76].

It is worth mentioning that content of bacterial endotoxin may influence the immune properties of chitosan. However, contamination with endotoxin from Gram-negative bacteria below 0.01EU/mg does not activate an immune cells. The influence of chitosan concentration, contamination with endotoxin, chemical modifications, presence of antigen or the rout of administration on immune and undesired effects are summarized in Table 3.

**Table 3:** The influence of different factors on chitosan-related immune effects and undesired reactions

Factor	Immune effect	Potential undesired reaction	Reference
Low chitosan dose	Anti-inflammatory (IL-1ra, type I IFN)	Minimal risk	[71]
High chitosan dose	Pro-inflammatory (IL-1β, PGE2 via inflammasome)	Local/systemic inflammation, tissue damage risk	[79]
Endotoxin contamination	Strong pro-inflammatory cytokine release	Severe inflammation, confounded safety profile	[80]
Presence of antygen	Enhanced adaptive immunity (Th1/Th2/Th17)	Hypersensitivity, DTH responses	[143]
Route (e.g., subcutaneous)	Depot effect, prolonged immune stimulation	Local inflammation, injection site reactions	[14]
Chemical modifications	Variable immunogenicity	Unknown, requires case-by-case evaluation	[212]

### Chitosan-based vaccines against bacterial pathogens

Emerging research has explored the immunomodulatory effects of chitosan, which could enhance its application in vaccine delivery by improving the immune response to various antigens [121]. Several studies have proposed chitosan nanoparticles or microparticles as carriers for numerous antigens, representing new candidate vaccines against bacterial infections. These nanoparticles are biocompatible, biodegradable, and non-toxic; have the desired size, shape, and large surface area; and exhibit high permeability and stability over a range of ionic conditions [226, 320, 321]. Hence, antigens loaded into chitosan carriers can be efficiently delivered to target sites. Additionally, chitosan derivatives can act as adjuvants, increasing the immunogenic properties of the vaccine antigens [179, 237, 238, 250].

The ideal nanoparticle size for vaccine applications generally ranges from 20 to 200 nm, particularly those around 50 nm, which often exhibit better uptake efficiency by APCs, such as dendritic cells or B lymphocytes [286]. Spherical shapes are common and effective in facilitating cellular uptake, while surface area plays a crucial role in antigen loading and delivery [322]. Although smaller particles are typically favored for uptake, larger ones (such as 160 nm) have demonstrated the ability to attract a greater number of immune cells to the injection site.

Chitosan's permeability and stability are significantly influenced by ionic conditions, particularly pH. Although chitosan is typically insoluble in water due to strong intermolecular hydrogen bonds, it becomes soluble in acidic solutions (pH < 6) due to the protonation of its amino groups. This protonation also influences its susceptibility to enzymatic degradation, with acidic conditions resulting in a more rapid breakdown. These changes in the properties of chitosan, which vary with pH, have significant implications for antigen delivery, as they can impact its ability to encapsulate and release antigens, as well as its interaction with biological tissues [304]. For example, a nanoparticle composed of chitosan could be designed to release antigen specifically at a targeted pH as in the stomach's acidic environment or lysosomes [15]. Chitosan's cationic properties at lower pH can enhance its interactions with negatively charged biological surfaces, potentially improving mucoadhesion (the adhesion to mucus membranes) or targeting [15, 91].

Chitosan-based vaccines against bacterial infections usually contain a protein or part of one, which represents the antigen or the DNA encoding such a protein. The latter is designed mainly to prevent infections spread by fish pathogens and was reviewed recently [6, 297]. Vaccines based on recombinant proteins require adjuvant systems to generate T helper (Th) 1-type immune responses. Chitosan is a good candidate in conjunction with IL-12, which induces T lymphocytes and NK cells to produce IFN-γ, granulocyte–macrophage colony-stimulating factor (GM-CSF), and TNF-α; directs CD4 + T lymphocytes toward Th 1 differentiation; and induces T-cell proliferation [104].

The regulation of immune cells, particularly macrophages and NK cells, is greatly influenced by the structure of chitosan and the content of endotoxins [71, 152]. Endotoxins, such as LPS, strongly activate the innate immune cells such as macrophages and NK cells via TLR4 while chitosan as another conservative pattern molecule via TLR2 and Dectin-1 [72]. Cytotoxicity of NK cells increased by chitosan may be an indirect effect driven by DCs, which deliver IFN-γ [211]. The activation of DCs by chitosan also prompts the release of different pro-inflammatory cytokines like IL-12 and IL-15, which further activate NK cells [72]. It has been shown that chitosan activates macrophages by stimulating the NLRP3 inflammasome, which triggers the release of pro-inflammatory cytokines such as IL-1β. It also initiates the STAT-1 pathway, producing pro-inflammatory cytokines and secretion of nitric oxide. However, chitosan can also influence the polarization of macrophages, steering them towards anti-inflammatory M2 phenotype that promotes tissue repair and alleviates inflammation but due to down regulation of the immune cells may promote the development of cancer [211, 213]. The content of endotoxins and the structure of chitosan are pivotal in influencing immune responses. Endotoxins primarily affect macrophage activation, while chitosan exhibits a broader range of effects that can engage both macrophages and NK cells. Understanding these interactions is essential for developing effective strategies for immune modulation and therapeutic interventions.

This section focuses mainly on protein-based chitosan vaccine candidates reported in the past five years, which are listed in Table 4.

**Table 4:** The list of the described chitosan-based vaccine candidates loaded with various proteins representing antigens

Pathogen	Infected organism	Vaccine matrix	Materials	Particles triggering immune response, loaded in chitosan	Vaccine distribution	Organisms in which in vivo* studies* were performed	References
Avian pathogenic *E. coli* (APEC)	Poultry	Ascorbate chitosan	Nanoparticles	Outer membrane protein-flagellar antigen (O-F)	Not specified	Chickens	[193]
*Bordetella bronchiseptica*	Mammals	Chitosan	Nanoparticles	Outer membrane vesicles (OMVs)	Subcutaneous	Rabbits	[161]
*Brucella abortus (B.abortus)*	Human	Chitosan	Nanoparticles	Malate dehydrogenase and/or outer membrane proteins (Omp10 and Omp19)	Intranasal	Mice	[261]
*B. abortus*	Human	Chitosan	Nanoparticles	Malate dehydrogenase	INTRANASAL	Mice	[262]
*Brucella melitensis (B. melitensis), B. abortus*	Human	Mannosylated chitosan	Nanoparticles	Flagellin FliC	Subcutaneous	Mice	[250]
*Campylobacter jejuni (C. jejuni)*	Poultry	Chitosan-sodium tripolyphosphate	Nanoparticles	Hemolysin co-regulated protein (hcp)	Oral	Chickens	[264]
*C. jejuni*	Human	Chitosan	Nanoparticles	Outer membrane vesicles (OMVs)	Oral	Mice	[265]
*Chlamydophila psittaci (Ch. psittaci)*	Animal	Chitosan	Nanoparticles	Multi-epitope peptide antigens	intramuscular and intranasal	Mice	[166]
*Ch. Psittaci*	Poultry	*Vibrio cholerae (V. cholerae)* ghost (VCG) – chitosan	Hydrogel	*Chlamydophila* elementary bodies (EBs)	Intranasal	Chickens	[158]
*Ch. Psittaci*	Poultry	*V. cholerae* ghost (VCG) – chitosan	Hydrogel	Multiple polymorphic membrane protein G (PmpG) antigens and major outer membrane protein (MOMP)	Intranasal	Chickens	[326]
*Clostridium perfringens (C. perfringens)*	Chicken (broilers)	Chitosan	Nanoparticles	Toxoids of extracellular proteins of *C. perfringens*, surface-tagged with *Salmonella* flagellar proteins	Oral	Chickens	[10]
Enterohemorrhagic *E. coli* (EHEC) *Shigella dysenteriae (S. dysenteriae)* type 1	Human	Chitosan	Nanoparticles	Chimeric recombinant protein: EIT comprising crucial immunogenic segments of EspA, intimin, and Tir of EHEC and two key virulence factors (STX1B-IPAD) of *S. dysenteriae*	Oral or injection	Mice	[197]
*Haemophilus influenzae (H. influenzae)*	Human	Mannose-modified chitosan	Microparticles	Nontypeable *H. influenzae* (NTHi) outer membrane protein P6	Intranasal	Mice	[179]
*Mycobacterium tuberculosis (M. tuberculosis)*	Human	Alginate-chitosan	Nanoparticles	PPE17, a surface localized protein displaying robust immunoreactivity in patients with active tuberculosis and represented as potent T-cell antigen and CpG	Intranasal or subcutaneous	Mice	[203]
*Salmonella* spp.	Chicken (broilers)	Chitosan	Nanoparticles	Outer membrane proteins (OMPs) and flagellin	Oral	Chickens	[62, 98, 232, 237]
*Salmonella enterica (S. enterica)*	Chicken (broilers)	Chitosan	Nanoparticles	Outer membrane proteins (OMPs) and flagellin	*In-ovo*	Chickens	[2]
*S. enterica*	Chicken (broilers)	Mannose-modified chitosan	Nanoparticles	Outer membrane proteins (OMPs) and flagellin	Oral	Chickens	[271]
*S. enterica*	Human	Chitosan	Nanoporous microparticles	Outer membrane proteins (OMPs)	Subcutaneous	Mice	[23]
*S. enterica*	Human	Chitosan	Nanoparticles	The tip protein of the *Salmonella* pathogenicity island 2 type III secretion system (SseB) fused with the LTA1 subunit of labile-toxin from enterotoxigenic *E. coli,* making the self-adjuvating antigen L-SseB	Intranasal	Mice rabbits	[58]
*Shigella flexneri (S. flexneri)*	Human	Trimethyl chitosan (TMC)	Nanoparticles	N-terminal region of IpaD antigen (NIpaD)	Oral	guinea pigs	[9]
*Streptococcus pneumoniae (S. pneumoniae)*	Human	Chitosan-maleimide	Nanocapsules	Pneumococcal surface adhesin A (PsaA)	Intranasal	-	[244]
*S. pneumoniae*	Human	Chitosan	Nanoparticles	Semisynthetic glycoconjugate (GC) composed of a synthetic tetrasaccharide mimicking the *S. pneumoniae* serotype 14 capsular polysaccharide (CP14) linked to the Pneumococcal surface adhesin A (PsaA)	Subcutaneous	Mice	[228]
*Streptococcus pyogenes (S. pyogenes),* group A streptococcus, GAS	Human	Trimethyl chitosan (TMC)	Nanoparticles	Lipopeptide with B-cell epitope J8 and T-helper epitope PADRE	Intranasal	Mice	[207]
*S. pyogenes*	Human	Amphiphilic chitosan derivative (arginine and oleic acid conjugated to the free amino groups present in the chitosan)	Nanoparticles	KLH protein (a source of T helper cell epitopes) and lipidated M-protein derived B cell peptide epitope (lipoJ14)	subcutaneous	Mice	[254]

The discussed vaccines are designed to prevent bacterial infections in humans, mammals or poultry. Among the vaccine candidates for humans, the proposed formulations are against non-typeable *H. influenzae* [179], *M. tuberculosis* [203],* S. pneumoniae* [228, 242] or *S. pyogenes*, also known as group A streptococci (GAS) [207, 254]. Another group involves bacteria responsible for life-threatening diarrhea in humans, such as enterohemorrhagic *E. coli* (EHEC) and *Shigella* spp. [9, 197], which are the leading causes of death in children under the age of five years worldwide [181]. Many of the described vaccines are linked to the prevention of diseases caused by zoonotic bacteria such as *Bordetella bronchiseptica* causing respiratory diseases in companion animals (mainly dogs and cats), or *Brucella* spp., which is often present in unpasteurized dairy products; poultry-origin *Salmonella* spp. [2, 23, 58, 62, 98, 161, 238, 270, 271], which is a major food-borne pathogen; the avian pathogenic *E. coli* (APEC) [193] *C. jejuni* [264, 265] and *Ch. psittaci* [158, 166, 326]*.* Vaccines against poultry-origin zoonotic pathogens have been designed for use in both humans and chickens to block bacterial colonization and prevent the transmission of pathogens to humans. One vaccine candidate was linked to a poultry disease, necrotic enteritis, caused by *C. perfringens* [10]. For some of these diseases, such as GAS-caused illnesses, no vaccines are currently available. In contrast, vaccines preventing pneumonia caused by *S. pneumoniae* are widely used in the clinic and have contributed to decreasing the number of pneumococcal infections worldwide. However, the available formulations offer protection to only some serotypes of *S. pneumoniae*, whereas the serotypes not included in the vaccine remain a threat to human health [228]. Finally, vaccines based on live attenuated bacteria or elementary bodies, for example, against *Ch. psittaci*, can cause disease in vaccinated animals [326] hence, new protein-based vaccines are needed, and promising candidates are presented in this section. Notably, in vivo studies were performed to determine the effectiveness of almost all proposed vaccines. In the case of human vaccines, animal models, such as mice, rabbits or guinea pigs, were used, whereas for poultry-dedicated vaccines, the model birds were chickens (Table 4).

Various proteins, usually recombinant proteins with immunogenic properties, have been loaded in chitosan as candidate vaccine formulations. The distinct outer membrane proteins (OMPs) were the most common, as summarized in Table 4. In many cases, such formulations contain several proteins, so-called protein cocktails, such as Omp10, Omp19, and malate dehydrogenase, in vaccines against *B. abortus* [262]. The use of protein cocktails can offer protection even from two different bacterial species, such as a vaccine against *S. dysenteriae* and EHEC, which is composed of two key virulence factors (STX1B-IPAD) of *S. dysenteriae* and a recombinant chimeric protein, rEIT, derived from crucial EHEC antigens (*E. coli* secretion protein A EspA), intimin, translocated intimin receptor (Tir)) [196]. Similarly, the fusion protein used in the vaccine against *S. enterica* contains the tip protein of the *Salmonella* pathogenicity island 2 type III secretion system (SseB) fused with the LTA1 subunit of labile toxin (LT) of enterotoxigenic *E. coli.* The LTA1 subunit is an adjuvant [58]. In some cases, the glycoconjugates or lipids were linked to proteins or peptides carrying the epitope. In a vaccine against *S. pneumoniae*, a synthetic tetrasaccharide that mimicked the capsular polysaccharide of *S. pneumoniae* serotype 14 was attached to pneumococcal surface protein A (PsaA) [228], whereas the vaccine against GAS contained peptides representing various epitopes linked with lipids [207, 254]. Interestingly, one report described formulations composed of a lipidated peptide conjugated to poly-L-glutamic acid (PGA), which, along with the lipid, functioned as an adjuvant. Moreover, the position of lipid attachment influenced the conformation of the peptide and the size of the produced chitosan nanoparticles [207]. Finally, larger structures, such as inactivated elementary bodies (EBs), were formulated in chitosan hydrogels as vaccines against *C. psittaci.* In contrast to live attenuated EBs, inactivated EBs are only marginally protective when used as vaccines. In the proposed formulation, the EBs were mixed with *V. cholerae* ghost (VCG) particles and a chitosan hydrogel solution, providing an effective adjuvant/delivery system [326]. VCGs represent empty bacterial cell envelopes lacking cytoplasmic contents and the cholera toxin [69]. Using inactivated EBs in VCG-chitosan improved their ability to trigger a protective immune response in chickens [227].

Notably, loading various antigens into chitosan often improves vaccine properties, as chitosan derivatives can act as adjuvants. A promising example is the vaccine against *Brucella* spp., where mannosylated chitosan improved the immune response to the vaccine [250]. In humans, the mannose residues present on antigens are recognized by specific mannose receptors located in the cell membranes of immune cells called APCs that trigger the activation of T lymphocytes. Hence, mannose residues in chitosan can also increase antigen presentation ability and improve the immune response [299]. Similar effects can be obtained by introducing groups that increase the positive charge of chitosan, such as the functionalization of chitosan with arginine, as was done in vaccines against GAS. The presence of arginine in the chitosan-based vaccine improved the interaction with APCs. In addition to arginine, chitosan was also modified with oleic acid; thus, the resulting chitosan derivative exhibited amphiphilic characteristics. Oleic acid has immunostimulant properties; hence, its introduction to chitosan further improved the immunogenic potential of the proposed vaccine [254]. The recent study by Zhao et al. reports the sucralfate acidified (SA) encapsulated N-2-hydroxypropyl trimethyl ammonium chloride chitosan (N-2-HACC) / N,O-carboxymethyl chitosan (CMCS) nanoparticles which were designed to work as adjuvant. In this model, bovine serum albumin (BSA) was used as an antigen. It was proved that such nanoparticles were highly stable in simulated gastric juice and intestinal fluid. The in vivo studies were performed in the guinea pig model. The vaccine was administrated orally and it significantly enhanced the residence time of BSA in the intestine up to more than 12 h. Additionally, it elicited the production of IgG and sIgA [322]. Notably, chitosan nanoparticles and emulsions were proposed as systems that can serve as adjuvants. Such an example is chitosan hydrochloride-stabilized Pickering emulsion (CHSPE) which was tested along with a lumazine synthase isolated from *Brucella* representing the antigen. CHSPE induced antigen-specific antibody levels, increased the ratio of central memory T cells (TCM) and effector memory T cells (TEM), and promoted the secretion of Th1-type cytokines. These effects were highly comparable to a commercial adjuvant ISA 206 [304].

Although some of the described chitosan-based vaccine candidates are intended to be injected subcutaneously, similar to many other vaccines used worldwide, the mucoadhesive properties of chitosan offer other routes of vaccine administration (Table 4). For instance, nasal immunization can trigger both systemic and mucosal immunity. The latter is the first line of defense against many pathogens. Additionally, it is non-invasive, as injections are not needed, and it requires a small dose of antigen. However, the main drawbacks of nasal vaccine administration are rapid clearance and inefficient uptake [312]. Chitosan-based vaccines can overcome these obstacles because of the mucoadhesive properties of chitosan, which prolong the retention of antigens at mucosal sites [299]. The intranasal route is a good choice for vaccines against pathogens such as *H. influenzae* [179] and *Brucella* spp. [62, 262], which usually infect their host via the mucosal surface or poultry-originating zoonotic bacteria such as *Salmonella* spp. [58] and *C. psittaci* [159, 326], which are present in aerosols originating from urine, feces, and other excretions of infected birds. One of the reports described the simultaneous intranasal and intramuscular administration of the vaccine against *C. psittaci*, which triggered strong humoral and cell-mediated responses in mice. Moreover, the intranasal application of chitosan nanoparticles improved the mucosal IgA response in the respiratory tract and reduced the bacterial load in the lungs [159, 166].

Chitosan-based vaccines can also be administered intragastrically or orally. This route is an especially good choice for bacteria that occur in the gut mucosa of birds, such as *C. jejuni* or *Salmonella* spp., and represents a safer alternative to vaccine injections, which slow the growth of the birds, cause tissue damage, are time-consuming and incur additional costs [62, 265]. Hence, the oral administration of vaccines is preferred in the poultry industry. The antigen is delivered to gut-associated lymphoid tissues, triggering a mucosal IgA response. Notably, a delivery system that protects antigens from degradation is required for efficient immunization via the oral route [299]. Chitosan-based vaccine nanoparticles administered by oral gavage in chickens reduced the degree of *C. jejuni* colonization in chicken intestines, consequently decreasing the risk of infection in humans [151, 265]. Another formulation against *C. jejuni* was tested in mice, and a similar effect was observed, with a significant reduction in the cecal bacterial load [264]*.* In addition to intranasal and oral routes of vaccine administration, vaccine candidates against *S. enterica* can also be delivered *in ovo* [2]. This type of vaccine is injected directly into the embryonic sac, and the antigens are swallowed by the embryo, inducing early immunity that results in successful protection from pathogens [82]. This method is cost-effective and represents another mass vaccination strategy for the poultry industry. Additionally, *in ovo* vaccination can replace stressful post-hatching procedures. In vivo studies on chickens have shown that *in ovo* vaccination with chitosan nanoparticles carrying antigens induces mucosal and systemic immune responses and does not cause hatchability loss [2].

In summary, chitosan and its derivatives have great potential as adjuvants and vaccine delivery systems. Nanoparticles are the most commonly proposed chitosan-based carriers for proteins or peptides triggering the immune response. Various chitosan modifications have been proposed to improve the material's properties. Finally, using chitosan opens the possibility of mass vaccination strategies involving intranasal, oral, or *in ovo* vaccine administration. Chitosan-based vaccine formulations offer numerous advantages over conventional adjuvants primarily due to its biocompatibility, biodegradability, and ability to enhance immune responses [311]. They induce less side effects than those containing classical adjuvants, such as aluminium salts or MF59 [87, 163]. Moreover, easy chitosan decomposition through natural processes in the human body results in its effective biodegradation and elimination. Chitosan nanoparticles provide several advantages over lipid nanoparticles (NPs) in delivery of vaccine antigens particularly in mucosal milieu. Compared to NPs, chitosan particles strongly attract mucus, enabling them to adhere to mucosal surfaces. They can also expand tight junctions of epithelial cells, allowing crossing of antigens through the mucosal barrier, which can promote the development of immune responses [296, 311]. However, LPNs seems to offer good solution for mRNA vaccines [276].

### Recent concepts for chitosan-based formulations against chronic infections and related diseases induced by the gastric pathogen *Helicobacter pylori*

*H. pylori*, a Gram-negative, microaerophilic bacterium that colonizes epithelial cells in the prepyloric part of the stomach in humans, is the main etiological agent of chronic inflammation of the gastric and duodenal mucosa, ulcers of these organs, and gastric cancer [51, 52, 180]. The WHO classified *H. pylori* as a class I carcinogen [53] The ineffective host immune response following *H. pylori* infection results from the ability of these microorganisms to weaken the functions of immunocompetent cells [13, 50, 51, 64, 90, 145, 210, 245]. The IgA antibodies developed against *H. pylori* at the mucosal level are not protective because *H. pylori* components inhibit the formation of secretory IgA dimers, and IgG antibodies are ineffective because of the location of the bacteria [199]. The increase in chronic *H. pylori* infections is caused not only by the ability of these bacteria to avoid the host's immune mechanisms but also by the development of drug resistance to commonly used antibiotics, such as clarithromycin, metronidazole, levofloxacin, amoxicillin, and tetracyclines [34, 88, 98].

The abovementioned difficulties in the treatment of *H. pylori* prompted the development of new formulations, including those with chitosan or modified chitosan, to support anti-*H. pylori* therapy and enhance the activity of immunocompetent cells. Owing to their mucoadhesive properties, ability to bind to *H. pylori* adhesins, and bactericidal properties resulting from the electrostatic interactions of the polymer with the bacterial cell wall, polymer-carriers based on chitosan have been proposed to support the treatment of *H. pylori* infections. The choice of chitosan as a material for the construction of the carrier is also due to its beneficial role in reducing the adhesion of *H. pylori* and its antimicrobial activity [84].

Recently, the ability of chitosan to inhibit urease production by *H. pylori* was demonstrated [43]. Luo et al. developed chitosan nanoparticles that exhibit the highest bacteriostatic activity at pH 4.0. Notably, they reported that chitosan nanoparticles with 95% deacetylation exhibited a stronger anti-*H. pylori* effect than those with 88.5% deacetylation [178]. In vitro studies have shown that chitosan microparticles are better able to bind live *H. pylori* at pH 6.0 than at pH 3.0. Moreover, at pH 6.0, a significantly greater affinity of chitosan microparticles for *H. pylori* strains positive for sialic acid binding adhesin (SabA) and negative for the blood antigen binding adhesin (BabA) than for BabA + /SabA- strains was demonstrated, with 76% and 50% reductions in adhesion, respectively [84]. Modifying microparticles by substituting glycans, such as Lewis b (Leb) and/or sialo-Lex, which specifically bind to *H. pylori* strains positive for BabA and/or SabA, also provides an opportunity for the development of personalized therapy [84]. The effectiveness of *H. pylori* eradication via the use of chitosan microparticles [106] or chitosan nanoparticles [178] was confirmed in vivo in a mouse model. Other researchers have proposed the encapsulation of chitosan NP transresveratrol (3,4,5-trihydroxystilbene), a flavonoid polyphenol present in grapes, to inhibit the growth of *H. pylori* and diminish *H. pylori*-driven oxidative stress as well as the inflammatory response in mice infected with this pathogenic bacteria [315, 316]. Fayed et al. used amoxicillin-loaded chitosan nanoparticles and inulin to specifically target the milieu of *H. pylori* infection, thereby minimizing the required dose and potentially preventing amoxicillin resistance. Amoxicillin-loaded chitosan nanoparticles displayed efficacy against *H. pylori,* with a half-maximal inhibitory concentration (MIC_50_) of 48.34 ± 3.3 ng/mL [73]. Khoshnoodet et al. encapsulated amoxicillin-docosahexaenoic acid in chitosan–alginate nanoparticles in vivo in a rat model and reported that *H. pylori* was eradicated in 21 days in animals receiving such nanoparticles at concentrations of 10 and 20 mg/kg [140].

Chitosan nanoparticles proposed by Lin et al. were modified with fucose and heparin, which enabled inhibition of *H. pylori* growth, and the addition of berberine increased the killing properties of chitosan itself [170, 171]. Another strategy was used by Yang et al., who obtained superparamagnetic iron oxide nanoparticles co-loaded with amoxicillin and chitosan/polyacrylic acid particles [305]. Inhibition of biofilm formation and destruction of the remaining structures was possible by covering chitosan nanoparticles with rhamnolipid, providing high loading capacity with clarithromycin or amoxicillin and stability (89% and 99%, respectively) in the acidic pH of the stomach [19, 20, 157]. Table 5 presents several studies describing the possible mechanisms of chitosan or chitosan modifications against *H. pylori*.

**Table 5:** Possible anti-*H. pylori* mechanism of chitosan and its modifications

Anti-*H. pylori* mechanisms	Chitosan/chitosan modification	References
Inhibition of urease production	Chitosan	[43]
Protection of drugs against stomach acid	Chitosan nanoparticles,	[170, 178]
Targeting lesions	Chitosan microspheres, Clarithromycin-loaded chitosan hybrid microsphere Hydrogel PH-sensitive chitosan hydrogels loaded with metronidazole Amoxicillin-loaded genipin-cross-linked fucose-conjugated chitosan/heparin nanoparticles	[3, 84, 169, 170]
Destroying the biofilm	Superparamagnetic iron oxide nanoparticles coloaded with amoxicillin and chitosan/ polyacrylic acid particles Clarithromycin-loaded chitosan hybrid microsphere Liposomes-polymer hybrid nanoparticles loaded with amoxicillin Rhamnolipids-chitosan hybrids nanoparticles loaded with clarithromycin	[3, 84, 305]
Changing the membrane’s permeability	Chitosan nanoparticles, PH-responsive chitosan-heparin nanoparticles	[171]

Mucoadhesiveness is an important property in the design of drug delivery systems against mucosal pathogens, including *H. pylori* [201]. The positive glucosamine residues of chitosan react with the negatively charged mucosal surface imparted by sialic acids and sulfuric acid esters. This process is preceded by the penetration and intertwining of the polymer with mucins and then the formation of weak chemical interactions, e.g., van der Waals forces or hydrogen bonds [84]. The increased adsorption of active compounds results from the ability of chitosan to damage cell-to-cell tight junctions in the epithelium [113]. In the case of orally administered vaccines, increased antigen exposure allows for more effective interactions with M cells [255]. Effective mucoadhesiveness is proportional to the degree of deacetylation (DD) and requires a low pH, at which chitosan takes the form of a base. This is a major limitation in the delivery of active substances after topical or systemic administration and causes the precipitation of chitosan in a neutral/basic environment. The solution to this problem involves trimethylated chitosan derivatives, which additionally increase the positive charge or the addition of the Pluronic®F-127 copolymer [201].

*H. pylori* antigens spread from the stomach and interact with innate immune cells in the small intestine; therefore, the release of chitosan or substances with antibacterial or immunomodulatory properties is expected in the proper niche of the gastrointestinal tract. One of the methods for obtaining a carrier insensitive to the acidic pH of the stomach that swells and releases bioactive substances at the alkaline pH of the intestine is the formation of polyelectrolyte complexes of chitosan with particles or other negatively charged biopolymers, e.g., alginate, heparin, and polyglutamic acid, which leads to the production of strong electrostatic bonds that are soluble in water. Another method involves cross-linking to form covalent bonds (e.g., with genipin or ethylene glycol) or ionic bonds (e.g., with pentasodium phosphate). However, enteric formulations can also be obtained using polymer coatings such as Eudragit®, cellulose, and acetic acid salts. This procedure protects the chitosan core against acidic pH owing to the presence of protonated carboxyl groups, which deprotonate at alkaline pH, resulting in swelling of the carrier and drug release. Moreover, their presence effectively increases drug encapsulation efficiency and reduces the drug release rate at acidic pH. Another possibility is to create chitosan nanocomposites by integration with a low concentration of graphene oxide, which ensures hydrophobicity and aggregation and hinders the release of the drug at acidic pH. At neutral pH, the chitosan composite becomes hydrophilic and thus can swell [66]. In the case of carriers dedicated to gastric diseases, the above methods and the adjustment of reagent concentrations can protect the three-dimensional structure of the carrier.

Work is still ongoing to develop therapeutic vaccines against *H. pylori* infection and to prevent infection in high-risk groups. In 2015, Xing et al. improved the former multiepitope vaccine CTB-UE, which consisted of a mucosal adjuvant, cholera toxin subunit B (CTB), and five cell epitopes from *H. pylori* urease, by using a chitosan‒CpG combination adjuvant to increase the immunogenicity of this epitope vaccine for oral immunization [300]. The results in a mouse model revealed that the levels of IgG2a, IgG1, and IgA in the serum and the levels of secretory IgA (sIgA) in the stomach, intestine, and feces were significantly greater in the vaccinated group than in the control group, which was not immunized. Moreover, chitosan–CpG combination adjuvants changed the IgG2/IgG1 ratio and promoted a Th1/Th17-dependent protective immune response. Additionally, Gong et al. reported that the use of chitosan instead of CT as an adjuvant in an anti-*H. pylori* vaccine resulted in higher anti-*H. pylori* antibody levels as well as higher IL-10 or IL-4 levels in mice and, moreover, increased the mRNA expression of TLR4 and decreased the number of CD4 + CD25 + Foxp3 + regulatory T lymphocytes, demonstrating that the use of chitosan as an adjuvant may drive therapeutic effects [88, 89].

#### Antiviral chitosan-based therapeutic formulations for HIV infection

Several studies have explored the potential of chitosan-based formulations for the delivery of drugs against HIV [146, 204, 205, 230]. Fonseca-Santos et al. developed chitosan-based nanoparticles for the delivery of anti-HIV drugs and demonstrated encapsulation efficiencies exceeding 70% and sustained drug release over 48 h [77]. Zhu et al. developed chitosan-coated liposomes for anti-HIV drug delivery, which showed superior stability with negligible drug leakage over 30 days [324]. The chitosan coating increased cellular uptake and prolonged drug retention. Chen et al. explored chitosan hydrogels for sustained anti-HIV drug release [72]. These hydrogels exhibited tunable drug release kinetics, with rates ranging from days to weeks, maintaining structural integrity under physiological conditions and thus ensuring prolonged drug availability. Chitosan-based microparticles developed by Wang et al. for anti-HIV drug delivery demonstrated high drug loading capacities exceeding 80%, with sustained drug release over 72 h [282]. The chitosan-coated nanofibers obtained by Liu et al. exhibited excellent biocompatibility and sustained anti-HIV drug release over 96 h [155]. The chitosan coating increased the stability and facilitated adhesion to biological surfaces, ensuring targeted drug delivery to HIV-infected cells. Cazorla-Luna et al. developed chitosan-based mucoadhesive vaginal tablets with mucoadhesive strengths exceeding 20 N and controlled release kinetics extending the controlled drug release of the anti-HIV drug tenofovir [40]. The tablets facilitated prolonged contact with the vaginal mucosa and controlled release of the drug for over 24 h, indicating the efficacy of chitosan as a carrier for sustained drug delivery. The formulation demonstrated excellent biocompatibility, with host cell viability exceeding 90%. Avlani et al. developed dispersible vaginal tablets of tenofovir loaded in mucoadhesive chitosan microparticles for anti-HIV pre-exposure prophylaxis, with mucoadhesive strength exceeding 25 N and controlled drug release over 48 h [22]. Additionally, the formulation demonstrated excellent biocompatibility, with cell viability exceeding 95%, underscoring the potential for safe and effective HIV prevention strategies. Singh et al. investigated chitosan and sodium alginate-based nanoparticles for anti-HIV drug delivery [266]. These nanoparticles exhibited drug encapsulation efficiencies exceeding 90%, and the controlled release profiles demonstrated sustained drug release over 72 h, indicating the potential for long antiretroviral therapy. Mishra et al. (2024) developed chitosan/sodium alginate hydrogels for sustained anti-HIV drug release [191]. The hydrogels displayed tunable drug release kinetics, with release rates ranging from days to weeks and excellent biocompatibility and stability under physiological conditions. Wang et al. explored the use of chitosan and sodium alginate microparticles for anti-HIV drug delivery [284]. The microparticles exhibited drug loading capacities exceeding 80% and a uniform particle size distribution. They also displayed sustained drug release profiles over 96 h, indicating the potential for prolonged therapeutic efficacy. Wang et al. investigated chitosan/sodium alginate composite films for anti-HIV drug delivery [284]. The films demonstrated excellent mechanical properties and biocompatibility with minimal cytotoxicity and exhibited sustained drug release over 48 h. Parth et al. developed chitosan/sodium alginate-based mucoadhesive hydrogels for anti-HIV drug delivery [216]. These hydrogels exhibited high mucoadhesive strength, ensuring prolonged retention within the vaginal mucosa, and the controlled drug release profiles extended over 72 h [129]. The nanoparticles demonstrated encapsulation efficiency exceeding 80%, ensuring optimal drug loading. The controlled release kinetics achieved sustained drug release over an extended period of time, up to 72 h. The PF-68-coated alginate nanoparticles exhibited excellent biocompatibility, with cell viability exceeding 90%, highlighting their suitability for safe and effective drug delivery applications in HIV treatment.

Notably, among antiviral therapies, similar to antibacterial therapies, vaccines are of great interest in global health procedures. Therefore, research on new vaccines or improvements to existing vaccines is fundamental. Zhang et al. conducted an interesting study on the development of chitosan-based vaccine formulations for enterovirus 71 (EV71), which is an etiological agent of gastric diseases in the Asia–Pacific region [313]. This study revealed that the oral immunization of female ICR mice with formulations containing recombinant VP1 protein and chitosan resulted in the induction of broad-spectrum immune responses, including VP1-specific IgA mucosal antibodies and specific IgG in the serum. Furthermore, splenocytes from immunized mice induced high levels of Th1, Th2, and Th3 cytokine responses related to IFN-gamma, IL-4, and transforming growth factor (TGF)-β, respectively. Hopefully, mucosal immunization via VP1, a major immunogenic capsid protein of the enterovirus EV71, may be a promising strategy to prevent EV71 infection.

In another study, Prego et al. proposed chitosan-based nanoparticles for improving immunization against Hepatitis B viral infection [229]. Chitosan-based nanoparticles allowed the efficient association of recombinant hepatitis B surface antigen (rHBsAg). This antigen was then released in vitro from the nanoparticles without compromising its antigenicity and was protected during storage at temperatures of 4 °C and − 20 °C. After intramuscular administration, these chitosan-based nanoparticles loaded with rHBsAg induced ninefold higher levels of specific antibodies than the conventional alum-adsorbed vaccine, confirming the adjuvant capacity of chitosan. These results show the potential of using chitosan to create new antiviral strategies based on vaccination.

#### Encapsulation of probiotics or other bacterial species in chitosan-derived health-promoting applications and the prevention of disease development

Chitosan is frequently used for the encapsulation of bacteria; however, it is usually used in combination with other biopolymers, of which sodium alginate is the most common, and chitosan is typically used as an outer layer, as shown in Fig. 2, to overcome the low resistance of sodium alginate to acidic pH in the stomach [145, 241]. Moreover, various additives, including organic compounds, polymers, or nanoparticles, are added to these particles to increase their stability under gastrointestinal conditions. One exception is nanofibers, in which bacteria grow in the voids between the net structure of those materials. Chitosan and chitosan oligosaccharides may also have beneficial effects on intestinal metabolism and the maintenance of eubiosis in both healthy and sick people by regulating the ratio of bacteria from the *Firmicutes* and *Bacteroidetes phyla* and promoting the growth of probiotic bacteria [49, 150].

The encapsulation of various probiotic bacteria, including *Lactobacillus acidophilus (L. acidophilus)*, *L. rhamnosus*, *L. casei*, *L. plantarum*, *L. reuteri*, *L. lactis*, *L. bulgaricus*, *L. salivarius* and *Bifidobacterium bifidum (B. bifidum),* for *per us* administration using different variants of chitosan-based carriers has been broadly studied in recent years. Microencapsulation of bacteria is used to enclose probiotic strains in the polymeric matrix and prepare foods that may have a beneficial effect on health. The encapsulated viable bacteria should be in the minimum range of 10^6^ to –10^7^ CFU/g to achieve the desired effect [175]. Examples of different bacterial species loaded in carrier formulations containing chitosan are listed in Table 6.

**Table 6:** Examples of different bacterial species loaded in carrier formulations containing chitosan

Bacterial species	Matrix	Materials and additions	Application	References
*L. acidophilus* NCIM 5306	sodium alginate, inulin, coated with chitosan	Microbeads	Probiotic (pomegranate juice)	[277]
*L. acidophilus*	gelatin (GE)–chitosan (CH) polyelectrolytes-coated with liposomes	Lipid-based nanocarriers	Probiotic	[4]
*L. acidophilus* *B. bifidum*	chitosan/sodium alginate	Microbeads	Probiotic	[12]
*L. acidophilus* *L. rhamnosus*	sodium alginate coated with chitosan and Eudragit S100	Microbeads	Probiotic (Doogh Beverage)	[225]
*L. rhamnosus*	chitosan/sodium alginate	Microgels	Probiotic	[319]
*L. rhamnosus*	sodium alginate coated with chitosan	Microbeads	Probiotic	[209]
*L. rhamnosus*	chitosan/sodium alginate	Macrobeads	Probiotic	[44]
*L. rhamnosus*	gum arabic/chitosan, gum arabic/trehalose/chitosan	Coacervates	Probiotic	[25]
*L. casei* 01	sodium alginate coated with chitosan	Microbeads	Probiotic	[272]
*L. casei*	chitosan, inulin	Microcapsules	Probiotic	[279]
*L. plantarum* F1 *L. reuteri* 182	sodium alginate coated with chitosan	Macrocapsules	Prebiotics	[124]
*L. plantarum*	gelatin–chitosan, and chitosan–gum Arabic	Coacervate microcapsules	Inhibition of colorectal cancer	[255]
*Lactiplantibacillus plantarum (Lactobacillus plantarum)* ZJ316	chitosan and pullulan	Nanofibers	Probiotic	[318]
*L. plantarum*	sodium alginate coated with chitosan	Microbeads	Probiotic	[174]
Lactic acid bacterium	sodium alginate coated with chitosan	Microcapsules	Prebiotics	[241]
*Lactococcus lactis (L.lactis)*	chitosan/sodium alginate	Microparticles	Probiotic	[68]
*L. bulgaricus*	sodium alginate coated with chitosan	Microcapsules	Probiotic	[111]
*L. salivarius*	chitosan/sodium alginate	Microcapsules	Probiotic	[306]
*Bacillus coagulans* (*B. coagulans*) NBRC-12583 *Enterococcus faecium (E.faecium)* MGFR1	sodium alginate, inulin, chitosan	Micro- and nano-particles	Probiotic	[83]
*P. fluorescens*	chitosan/sodium alginate	Microbeads	Biofertilizer	[28]
*Paenibacillus. polymyxa (P. polymyxa)*	chitosan/carrageenan	Macrobeads	Biofertilizer	[131]
*E. coli*	chitosan/alginate and chitosan/dextran sulfate	Film	Encapsulation	[217]
*Streptococcus salivarius S.salivarius)* LAB813	sodium alginate coated with chitosan	Microcapsules	Probiotic	[54]
*Streptomyces fulvissimus (S.fulvissimus)* Uts22	chitosan, gellan gum, zinc nanoparticles	Microcapsules	Biofertilizer	[247]
*B. bifidum*	sodium alginate coated with chitosan	Microbeads	Probiotic	[307]
*Mycobacterium bovis* (*M. bovis*) BCG	chitosan/pluronic	Microparticles	Treatment of *H. pylori* infection	[85]

#### *Lactobacillus acidophilus* and *Lactobacillus rhamnosus*

*L. acidophilus* has been encapsulated in microparticles to prepare pomegranate juice [277], yogurt [12], and beverages [225]. The extrusion method was used to obtain the microparticles added to the juice, and the particle size ranged from 190 µm to 260 µm. Four different formulations were developed: (a) sodium alginate beads, (b) sodium alginate + 1% inulin-containing beads, (c) chitosan-coated sodium alginate beads, and (d) sodium alginate + 1% inulin and chitosan. The highest survival rate of *L. acidophilus* was shown for inulin-containing beads and inulin-containing beads coated with chitosan [272]. *L. acidophilus* was enclosed in the sodium alginate core and covered with chitosan in the probiotic yogurt. Higher survival of bacteria during the storage of the final product [12]. The viability of *L. acidophilus* and *L. rhamnosus* was even greater in sodium alginate macrobeads covered with chitosan and Eudragit S100 nanoparticles with approximately 100–150 nm sizes. The presence of Eudragit S100 nanoparticles ensures bacterial release at the colon zone. A recent paper by Adeel et al. described the encapsulation of *L. acidophilus* in gelatin (GE)–chitosan (CH) polyelectrolyte-coated nanoliposomes [4]. However, since the typical size of those bacteria is 0.6–0.9 μm in width and 1.5–6.0 μm in length [35], encapsulating such objects in nanoliposomes with sizes ranging from 130 to 430 nm is impossible.

An alginate/chitosan microgel loaded with *L. rhamnosus* significantly decreased the degree of hepatorenal injury caused by salt stress in the mice. The viability of encapsulated bacteria was greatly improved over that of free bacteria after exposure to 2% bile salt [319]. Similarly, *L. rhamnosus* was encapsulated in sodium alginate coated with various materials to increase its stability under gastrointestinal conditions. The alginate/chitosan particle size was approximately 200 µm; however, the loading efficiency was lower than that of alginate/xanthan gum; therefore, only those materials were further investigated [209]. Moreover, alginate/chitosan (AC) capsules were compared with double bilayer AC (ACAC) capsules. Layer-by-layer (LbL) mixing was employed to prepare the desired particles, and their size ranged from 1 to 3 mm. The stability of the cells in the simulated gastric and intestinal fluid was greatly enhanced after encapsulation in the ACAC capsules. Moreover, the bacteria release was more sustained for ACAC capsules than AC capsules in the intestinal simulation solution (pH 6.8). In contrast, there was no release in the simulated stomach environment (pH 1.8) [44]. The interactions of oppositely charged polyelectrolytes were also used to construct coacervates composed of gum Arabic, chitosan, and sodium tripolyphosphate with the addition of trehalose, which significantly increased stability during storage and stress conditions [25].

#### *Lactobacillus casei*

Gel capsules were designed to encapsulate *L. casei* and contained various substances, including sodium alginate covered with chitosan. This system enhanced the viability of bacteria compared to free cells; however, minimal loss of bacterial survival over the digestion period was observed [272]. Combining inulin and chitosan was subsequently used to encapsulate *L. casei* and anthocyanins. Two populations of particles were observed, one whose size ranged from 63 to 97 μm and the second consisting of large coacervates with sizes up to 190 μm. Most importantly, the survival rate of bacteria was 98% in simulated gastric and intestinal juices. Additionally, the inhibitory activity of the particles toward α-glucosidase and α-amylase indicates the potential of this formulation for the treatment of postprandial hyperglycemia. Therefore, the obtained formulations (2% and 5%) were added to soft cheese and compared with the blank formulation. After encapsulation, high bacterial viability was achieved in experimental cheese samples, along with anthocyanin release in simulated intestinal juice [279].

#### *Lactiplantibacillus plantarum (L. plantarum) *and *Lactobacillus reuterI*

Macrobeads of sodium alginate were prepared via the extrusion technique subsequently coated with chitosan or supplemented with inulin and/or trehalose, and used to encapsulate *L. plantarum* and *L. reuteri.* The alginate/chitosan macrobeads showed the best encapsulation effect because of ionic crosslinking between those macromolecules. During 365 days of storage, the highest survival rate of encapsulated bacteria in such formulations was observed after lyophilization, and the most reasonable duration of storage was 180 days [124]. Moreover, alginate macrobeads with inulin and chitosan coatings can be prepared by electrospraying a double emulsion (W_1_/O/W_2_) composed of a water phase and sunflower oil as the organic phase. The addition of inulin enhanced bacterial viability; however, the presence of chitosan slightly decreased bacterial viability, which is in agreement with other findings [310]. Moreover, the lowest reduction in the viability of bacteria under gastric conditions was shown for chitosan-coated microbeads because of the decrease in the porosity of the microbeads caused by the presence of chitosan on their surface. A different approach was proposed by Yazdani and Rafiei et al., who used coacervate microcapsules composed of gelatin–gum Arabic, gelatin–chitosan, and chitosan–gum Arabic for the encapsulation of *L. plantarum* combined with omega-3 fatty acids (Omega-3) [257]. This study aimed to treat colorectal cancer (CRC) and achieve a synergistic effect of probiotics and omega-3 fatty acids. A high encapsulation efficiency of omega-3 was achieved, accompanied by a lower encapsulation efficiency for bacteria. As a proof of concept, an MTT assay on the C26 cell line was performed, which revealed that gelatin–chitosan has the greatest potential. However, loading these substances in the interior of the particles did not increase their efficiency, as tested by the expression levels of the bcl-2-like protein 4 (BAX) and caspase 3 genes in C26 cells. The substances were not tested under gastric conditions, and the size of the particles does not allow their uptake by C26 cells. Hence, the interference is due only to releasing the encapsulants that hinder the desired effect.

**Fig. 3:**
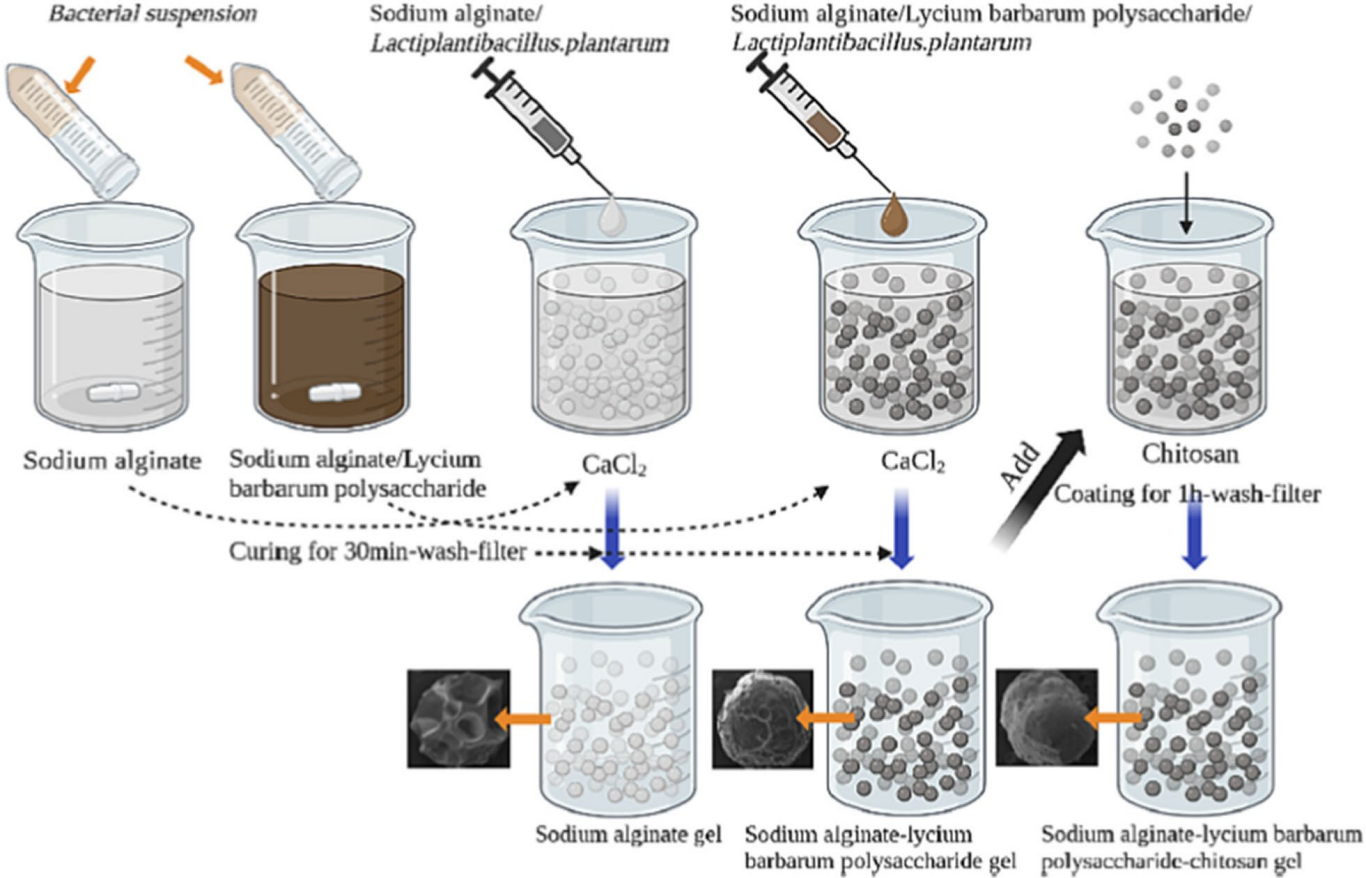
Scheme of the encapsulation process of the *L. plantarum* SHS01 using sodium alginate (SA) solution and *L. barbarum* polysaccharide (LBP) solution as encapsulating material, coated with chitosan obtaining sodium alginate-*L. barbarum* polysaccharide-chitosan gels (SLCG), with SA: LBP = 1:2,1:1, 2:1. (Copyright Elsevier, with the permission no. 5884130839175 of the publisher)

Nanofibers composed of chitosan and pullulan were proposed for encapsulating *Lactiplantibacillus plantarum (L. plantarum)* ZJ316. However, these bacteria are 0.9–1.2 μm wide and 3–8 μm long, so encapsulation is impossible [38]. Therefore, it may be assumed that bacteria covered the surface of the nanofibers or colonized the voids between them. This explanation is probably not adequate for the large difference between “encapsulated” and free cells in gastric (87 vs. 79%) and intestinal (79% vs. 69%) conditions [318]. In addition, chitosan was used as a shell to prepare sodium alginate/*Lycium barbarum* polysaccharide as the core of microbeads loaded with *L. plantarum*. The preparation methods and morphologies of the obtained particles are summarized in Fig. 3. Three different formulations were prepared: sodium alginate alone, sodium alginate/*L. barbarum* polysaccharide, and sodium alginate/*L. barbarum* polysaccharide coated with chitosan. The viability of bacteria and their thermal and storage stability was greater in the presence of chitosan on the bead surface and its hydrogen bond interactions with polysaccharides. The release of probiotics from chitosan-coated microbeads reached 88.16%, with a 9.657 log CFU/mL level after 5 h [174].

#### *Lactococcus lactis*

An external ionic gelation procedure was employed to prepare alginate-based microparticles loaded with *L. lactis*. These microparticles were subsequently coated with chitosan and Eudragit® RS 100; moreover, formulations with various adjuvants (methylcellulose, carboxymethylcellulose, carbopol, β-cyclodextrin, starch, carrageenan) were also prepared. The obtained particles possessed irregular shapes ranging from 200 to 400 µm. The level of encapsulated bacteria met the required criteria, and their release occurred in 30 min for almost all formulations; however, it was the slowest for alginate covered with chitosan at 60 min. Microparticles with carboxymethylcellulose and methylcellulose presented the highest stability in both simulated gastric and intestinal juices. These formulations were the best for long-term storage; however, the results for alginate/chitosan were also satisfactory [68].

#### *Lactobacillus bulgaricus*

Ionic gelation and internal emulsification were used to load alginate/chitosan microparticles with *L. bulgaricus*. The encapsulation efficiency of bacteria was high, as was their viability in gastrointestinal juice. The probiotic properties of these compounds were tested via dilatory administration to Beluga fish (*Huso huso*). The fishes were divided into different groups: the first group received microparticles loaded with bacteria; the second and third groups received bacteria-loaded microparticles and a basal diet; the fourth group received a basal diet and free bacteria. Significantly, the diet with bacteria-loaded microparticles activated granulocytes, assessed based on enhanced cell nitro blue tetrazolium (NBT) reduction activity. Globulin levels and hematological parameters also increase [111].

#### *Ligilactobacillus salivarius* (*L*. *salivarius*)

A different approach was proposed by Li et al., namely, the layer-by-layer microencapsulation of *L. salivarius* Li01 [306]. First, the bacteria were covered with carboxymethyl chitosan, and subsequently, alginate was crosslinked with metal ions to form bilayers with a maximum of three layers; nevertheless, two bilayers were sufficient to cover a single bacterial cell with a biopolymer shell [93]. As expected, the viability of bacteria in such shells was increased in simulated gastric juice and intestinal fluids. To investigate the effects of this approach for treating inflammatory bowel disease or DSS-induced colitis in mice, the animals were treated with encapsulated or free bacteria. Compared with free bacteria, encapsulated bacteria had a superior ameliorative effect, resulting in faster recovery of body weight and higher overall survival.

#### *Bifidobacterium bifidum*

The extrusion method was used to prepare microbeads loaded with *B. bifidum* sodium alginate; subsequently, those particles were covered with the first layer of chitosan and a second layer of whey protein. These particles were added to yogurt; however, there was no significant difference in the viability of single- or double-coated bacteria after 28 days of storage. Nevertheless, the viability of free bacteria decreased significantly, indicating that encapsulation is necessary to increase the stability of bacteria during storage. The antagonistic effects of free and encapsulated bacteria against *Salmonella spp*. were investigated via an agar diffusion assay. Free bacteria have an inhibitory improved impact compared with encapsulated bacteria; moreover, the antibiotic used as a control was more effective than the bacteria. Nevertheless, the probiotic effects of these microbeads have been proven, and in combination with antibiotics, their effects may be more pronounced [307].

#### *Bacillus coagulans* NBRC-12583 and *Enterococcus faecium* MGFR1

Chitosan–alginate nanoparticles were prepared by enclosing *B. coagulans* NBRC-12583 or *E. faecium* MGFR1 in an alginate, chitosan, and inulin matrix. Four formulations were prepared: (1) *B. coagulans* in chitosan, alginate, and inulin (BC Chit-Alg); (2) *E. faecium* in chitosan, alginate, and inulin (EF Chit-Alg); (3) *B. coagulans* in chitosan-alginate nanoparticles containinginulin (BC Chit-Alg Nano); (4) *E. faecium* in chitosan-alginate nanoparticles containing inulin (EF Chit-Alg Nano). The highest survival rate of bacteria in provoked gastric and intestinal fluids was shown for EF Chit-Alg Nano [83].

#### *Pseudomonas fluorescens*

Chitosan and sodium alginate were used as a matrix to encapsulate *P. fluorescens* bacteria and release them to increase crop productivity. Interestingly, chitosan was a more efficient matrix than sodium alginate for enhancing shelf-life; moreover, the viability of bacteria remained high even after ten months. The obtained chitosan particles degraded within 15 days and started to rupture after 20 days, releasing the bacteria into the soil. Moreover, a sustained release of the bacteria was achieved, lasting for 18 days. The zero-order model was used to describe the constant release of bacteria over time, independent of the amount of encapsulated *P. fluorescens*. These particles were used as biofertilizers in tomato crops to test their efficiency. After treatment with chitosan-based beads, significant nutrients (N, P, and K) increased, and the fungal count in the product decreased. Therefore, these particles are an effective and environmentally friendly alternative to chemical fertilizers [28].

#### *Paenibacillus polymyxa*

Chitosan–carrageenan macrobeads with different ratios of the two macromolecules were prepared via an ionotropic gelation method for the encapsulation of *P. polymyxa* and used to fertilize pak choi and Chinese cabbage. Interestingly, the bacteria released in the PBS and soil mixtures were the most abundant for carrageenan particles (chitosan/carrageenan ratio of 1:1). Most importantly, the use of macrobeads protects plants from clubroot disease and, as a result, increases plant height and shoot fresh weight. This was explained by the protective effects of the bacterial matrices against environmental conditions, combined with sustained release. Therefore, this approach is a green and scalable method for preparing plant growth-promoting macrobeads as biofertilizers [131].

#### *Escherichia coli*

The LbL encapsulation process consists of covering a negatively charged bacterial surface with oppositely charged ion pairs composed of a polycation (chitosan) and a polyanion (alginate or dextran sulfate). Zeta potential measurements indicated that charge reversal can be achieved by coating with chitosan, resulting in a positive surface charge. In contrast, the negative charge is recovered after the addition of alginate. This process can cover bacteria with multiple layers of polyelectrolyte pairs. It was used for covering *E.coli*. The bacteria were alive after the coating and the lag phase and entered the classical growth phase [217].

#### *Streptococcus salivarius *LAB813

Microbeads of alginate (4.1 to 6.6 mm diameters) covered with chitosan were used to encapsulate *S. salivarius* LAB813 but such encapsulation allows bacteria to survive for up to 5 days. Compared with noncoated capsules, coated capsules exhibited increased antimicrobial effects even at elevated temperatures [54].

#### *Streptomyces fulvissimus* Uts22

The spray-drying technique was used to encapsulate *S. fulvissimus* Uts22 and ZnO nanoparticles in combination with chitosan and gellan gum (3:1 ratio). Microparticles 140–150 μm in size were obtained, and nanoparticle release was sustained for 60 days. Finally, the effectiveness of these particles in enhancing plant growth (wheat plants) was shown in a greenhouse experiment. Encapsulation increases the efficacy of bacteria in managing plant diseases and facilitates the attainment of the highest fresh weight of roots and shoots. This combination can benefit the agricultural industry [248].

#### *Mycobacterium bovis* BCG

The spray-drying method was employed to fabricate microparticles loaded with *M. bovis* Bacillus Calmette-Guerin (BCG). The immunomodulatory properties of *M. bovis* BCG are of interest for developing carrier formulations for treating infections caused by the *H. pylori* gastric pathogen. Three formulations were prepared: chitosan alone, chitosan with N-acetyl-d-glucosamine, or chitosan with Pluronic F-127. The highest viability of encapsulated bacteria was observed for particles with Pluronic F-127. Interestingly, the release pattern of bacteria differed among the prepared formulations. Those composed solely of chitosan or chitosan/N-acetyl-d-glucosamine released mycobacteria under acidic conditions, whereas those composed of chitosan/Pluronic F-127 released bacteria only at physiological or alkaline pH. This finding implies that the release of *M. bovis* BCG can be controlled by the selection of the matrix. If so, the choice of the matrix will facilitate the delivery of the bacterial cargo to the stomach or the gut. The obtained particles were safe for various cell models (L929 mouse fibroblasts, guinea pig primary gastric epithelial cells or fibroblasts, and human THP-1 monocytes) and guinea pigs in vivo. However, the effectiveness of microparticles loaded with *M. bovis* BCG against experimental *H. pylori* infection should be confirmed [85].

## Chitosan-derived biomaterials for regenerative medicine

### Potential applications in bone and cartilage tissue regeneration

The natural process of osseous tissue regeneration takes a long time, and the newly formed bones have less strength. In clinical practice, treating large-size cell structure defects usually involves bone transplantation; however, these procedures have many disadvantages. An alternative is using bone tissue substitutes, such as scaffolds, in tissue engineering [319]. The human body should recognize the best scaffolds supporting tissue reconstruction as safe and mimic the natural microenvironment, facilitate cell adhesion, and provide optimal metabolic conditions. This is possible by mixing chitosan with other natural or synthetic polymers and/or bioactive molecules [292]. As demonstrated in both in vitro and in vivo studies, chitosan-composites enriched by growth factors including bone morphogenetic proteins (BMPs) e.g. BMP-2 and BMP-7, vascular endothelial growth factor (VEGF), essential fibroblast growth factor (bFGF) or peptides that mimic their function promotes cell migration, proliferation, differentiation of mesenchymal stem cells (MSC) into osteoblasts, angiogenesis and generally improve new bone formation [192, 325].

Large doses of drugs are required to repair bone defects caused by tuberculosis, osteoporosis, osteosarcoma, or osteomyelitis. The in vitro study showed a sustained and controlled release of the anti-tuberculosis drugs (i.e., rifampin and isoniazid) from hydroxyapatite-chitosan composite [8]. Similar observations concerned the release of resveratrol during therapy of osteoporotic rat femur bone defect [156], cisplatin [269] or doxorubicin [81] in osteosarcoma-affected bones and antibiotics in osteomyelitis [274, 275]. In addition, using chitosan-composite with resveratrol down-regulated the expression of inflammatory markers, TNF-α, IL-1β, and iNOS during osteoporosis therapy [156]. This evidence indicates the potential use of chitosan in bone implant-associated infections and other bacterial diseases of bony tissue and lays the groundwork for future exploration.

The wide range of possible uses of chitosan is not only a consequence of its specific properties but also results from mixing it with different components and forming, e.g., into films, hydrogels, nanofibers, microspheres, tablets, pastes, 3D-printed scaffolds. Its wide application in dentistry deserves special attention. Chitosan is used as a material for the production of implants and enables bone regeneration around implants [21]. Mesenchymal stem cells with chitosan were implemented in clinical settings for alveolar bone augmentation. It could be used as a hemostatic agent during dental surgery to promote shorter bleeding time thanks to electrostatic interaction with erythrocytes and blood plasma [21, 153]. Chitosan has been found to have a remineralizing property and play an essential role in enamel and dentin regeneration [21]. Its antibacterial and antifungal effect on microorganisms is associated with developing dental plaque and oral inflammation [21]. This property can enhance their bacteria-fighting ability as a carrier for antibacterial drugs. Drug delivery systems based on chitosan-combined compounds are widely used in treating dental caries, periodontitis, dental pulp regeneration, nonsurgical treatment of residual periodontal pockets, and prolonged anesthesia [118, 184]. In addition to anti-caries drugs, it can protect nucleic acid (like DNA) and effectively transport anti-caries vaccines. The comprehensive effect of chitosan-based biomaterials is the reason for its different regenerative applications.

As with skeletal tissue, traumas and other inflammatory and degenerative cartilage damages are treated by various methods. Available therapeutic strategies often have a poor effect and are not free from adverse events, so a new cartilage repair approach is needed. Due to the properties above of chitosan and especially its structural similarity to the glycine aminoglycan (common in connective tissue), this polymer can also be helpful as a scaffold in regenerative cartilage therapy. Osteoarthritis causes increased mortality of cartilage and chondrocytes, leading to chronic pain and disability, and there is no effective treatment for this disease yet [137]. In the in vivo studies, intra-articular injection of hydrogel based on chitosan enriched with, among other things, modified hyaluronic acid (HA) significantly reduces inflammation in comparison to traditional HA injection [198]. The results of studies in animal models of osteoarthritis indicate that some compounds, e.g., lectin, and lactose, in combination with chitosan, reduce the levels of TNF-α, IL-1β, IL-6, IL-17, rheumatoid factor, reactive oxygen species, and C-reactive protein [97, 168, 198]. The reduction of disease symptoms was confirmed in a study involving patients with age-related cartilage degeneration, including osteoarthritis [186, 294].

### Potential applications in soft tissue engineering

In the context of chitosan-based soft tissue engineering, mesenchymal stem cells (MSCs) are frequently utilized due to their multipotency and capacity to differentiate into various cell lineages, including myogenic pathways. These MSCs are typically harvested from adult tissues such as bone marrow or adipose tissue. Their integration with chitosan-based scaffolds has shown promise in facilitating tissue regeneration.​ [185]. Chitosan, a biopolymer derived from the deacetylation of chitin, possesses several properties that make it suitable for scaffold fabrication in tissue engineering. Its biocompatibility, biodegradability, and structural similarity to glycosaminoglycans enable it to mimic the extracellular matrix (ECM), thereby supporting cell adhesion, proliferation, and differentiation. When utilized as a scaffold, chitosan can be engineered into various forms, including hydrogels, nanofibers, and porous structures, to suit specific tissue engineering applications [141, 167, 172].

Regenerative medicine involves the repair/regeneration of soft tissues such as muscles, nerves, and skin. This is particularly important in the case of tissues with extremely low regenerative capacity, such as heart muscle cells. For example, cardiovascular diseases may lead to myocardial infarction, leaving an area of cell necrosis. Chitosan-based polymers are modified with other polysaccharides, proteins, or biodegradable polymers to imitate the properties of native myocardial tissue best. Stem cells are introduced into chitosan-based three-dimensional scaffolds applicable to infracted tissue. Chitosan scaffolds stimulate myoblasts' proliferation and differentiation and improve the heart muscle's or damaged arteries' regenerative properties [16, 26, 136]. Most likely, mechanically and electrically stimulating the stem cells in the scaffolds affects the VEGF family's glycoprotein receptors, e.g., VEGF-A, that induce cardiovascular regeneration by increasing the capillary density [26]. The effect of the same biopolymers, including chitosan, was investigated in animal models with induced injury or volumetric muscle loss, and the obtained results are consistent with those described for myocardial muscle [6].

In the context of myoblast stimulation, chitosan-based scaffolds have demonstrated the ability to enhance myogenic differentiation. This is attributed to their capacity to adsorb and retain growth factors and cytokines, creating a conducive microenvironment for myoblast proliferation and maturation. Additionally, the mechanical properties of chitosan scaffolds can be tailored to match those of native muscle tissue, providing the necessary cues for myogenic cells. Studies have shown that chitosan–gelatin composite scaffolds, in particular, exhibit mechanical properties similar to muscle tissues and support the growth and differentiation of myoblasts [96].

Furthermore, the incorporation of bioactive molecules and the functionalization of chitosan scaffolds can further enhance their efficacy in muscle tissue engineering. For instance, the addition of specific peptides or the modification of surface properties can improve cell-scaffold interactions, leading to improved outcomes in tissue regeneration. ​

In nerve regeneration, research focuses on assessing the effect of using polymer-based tubular channels in more significant (more than 3 cm) nerve gaps in the peripheral nervous system. These implanted tubular conduits are polymeric tubes with various fillers in combination with stem or somatic cells. Biochemical fillers make it possible to create a favorable environment for axon regeneration, while physical fillers mimic the complex architecture of the nerve. Used together, it not only protects the injured nerves but also leads to an increase in the surface of the internal canal, providing optimal conditions for the adhesion and growth of nerve cells, as well as the formation of myelin sheaths. When chitosan nanofibers and chitosan composite fibers were used as a filler, an increase in the regeneration rate of the peripheral nerves, like the sciatic nerve, was observed [273, 314]. Using chitosan as hydrogel-based scaffolds may be promising for regenerating nerves after spinal cord injury [298]. However, hydrogels have the most excellent prospects in wound dressings. Conventional hydrogels are constantly modified, enriched, and evaluated to develop new multifunctional dressings with better parameters. Due to their structural similarity to soft tissues, high water content, elasticity, and all other beneficial properties of chitosan-based hydrogels, they have been widely used in both preclinical and clinical treatment of wounds after radiation and/or burn injury [258], after skin melanoma surgery [42], in chronic skin ulcers [278], in diabetes and in the treatment of other infected wounds.

The results mentioned above indicate the high potential of chitosan-based scaffolds and hydrogels as a less invasive alternative in the regeneration and treatment of bone, cartilage, nerve, and soft tissue damage. Further refinement of the composition and architecture of hydrogels/scaffolds is needed to expand existing applications to new diseases.

#### Application of chitosan formulations in cancer treatment

Chitosan's primary advantage in cancer treatment lies in its ability to enhance the delivery and efficacy of chemotherapeutic agents. Its cationic nature allows for effective binding with negatively charged molecules, including DNA and proteins, facilitating the encapsulation of drugs that can be selectively released at tumor sites. This targeted delivery minimizes the systemic side effects typically associated with chemotherapy and maximizes therapeutic impact at the disease locus. Furthermore, chitosan's properties can be adjusted through chemical modifications, thus tailoring its solubility, degradability, and interaction with cellular membranes. These modifications enhance its potential as a carrier in drug delivery systems, enabling it to bypass cellular barriers and release drugs directly into the cytoplasm of cancer cells. As discussed above, chitosan and its derivatives also show immunostimulating properties, which can boost the body's immune response against cancer cells.

The tumour microenvironment (TME) comprises many types of immune cells, including macrophages, neutrophils, lymphocytes, NK cells, DCs, and bone marrow-derived suppressor cells. Chitosan causes beneficial changes in the expression patterns of pro-inflammatory cytokines, which activate tumour-antagonizing immune cells playing critical roles in recognizing and destroying tumour cells. Chitosan can activate dendritic cells to express IL-12 and IL-15, which in turn activate the STAT4 and NF-κB signaling pathways, respectively, in NK cells. [159]. Additionally, chitosan may initiate the polarization of M1 macrophages that are anti-tumor by producing pro-inflammatory cytokines and reactive oxygen/nitrogen species and reducing the immunosuppressive properties tumor microenvironment [61]. Notably, the need to increase the immunogenicity of cancer antigens derived from the host’s cells is emphasized. These effects could be achieved by using chitosan as an adjuvant, as Li et al. (2013) and Lu et al. (2024) have widely discussed [159, 176, 177].

Among immune cells in TME, tumor-associated macrophages (TAMs), tumor-associated neutrophils (TANs) and regulatory T lymphocytes (Tregs) are classified as tumor-promoting immune cells [152, 177]. TAMs may have not only the M1 phenotype, but also anti-inflammatory M2 phenotype, which can promote tumor development due to inhibition of the host immune responses. Chitosan as an component of nanoparticles containing compounds with antiangiogenic and antiproliferative effects will additionally support their action by inducing repolarization of the tumor-supportive M2 macrophages to the tumor-suppressive M1 macrophages [149]. Similarly, chitosan may promote a balance between the pro-inflammatory (N1) and regenerative (N2) phenotype of neutrophils, which will support the anti-tumor effect of the therapy [177]. However, determining the role of neutrophil subpopulations in the tumor development in conjunction with chitosan on this process still requires clarification.

This review will discuss the application of chitosan formulations in cancer treatment based on gastrointestinal cancers. Some examples of other anti-cancer experimental therapies will also be included. In colon cancer, chitosan nanoparticles have been investigated for their ability to deliver chemotherapeutic agents directly to the colon, thus reducing the systemic toxicity of the drugs. Chitosan's mucoadhesive properties ensure that the nanoparticles are retained longer in the colon, allowing for sustained release of the encapsulated drug. This approach has shown promise in increasing the efficacy of drugs like 5-fluorouracil, a commonly used chemotherapeutic agent in colon cancer treatment [280].

Developing oral formulations for colon cancer treatment utilizing chitosan nanocomposites represents a significant advancement in oncologic pharmacotherapy. Chitosan's bioadhesive nature allows these nanocomposites to adhere to the mucosal lining of the gastrointestinal tract. Encapsulating anticancer drugs within chitosan nanocomposites protects the active ingredients from the stomach's acidic environment and the enzymatic degradation in the upper gastrointestinal tract. This ensures that the drug remains intact until it reaches the colon. Moreover, chitosan’s ability to control the release rate of the encapsulated drug optimizes the therapeutic efficacy by maintaining the required plasma concentration of the drug for an extended period [55].

Specific targeting of colon cancer cells can be achieved by modifying chitosan nanocomposites with ligands that recognize and bind to receptors overexpressed on the surface of colon cancer cells. This receptor-mediated targeting enhances the selectivity of the drug delivery system, reducing the systemic side effects typically associated with conventional chemotherapy. Chitosan facilitates the opening of tight junctions between epithelial cells, increasing paracellular permeability and enhancing the drug's transmucosal transport. This property benefits oral drug delivery systems, where epithelial barriers can significantly hinder absorption. The use of oral chitosan nanocomposites for colon cancer treatment improves patient compliance due to the ease of administration and enhances the pharmacokinetic profile of drugs. The localized drug release in the colon reduces the required dosage and frequency of administration, minimizing potential adverse effects. In 2021, they were encapsulated in chitosan nanoparticles oxaliplatin and resveratrol as a possible new therapeutic strategy for colorectal cancer therapy. The inhibition efficiency and anti-colon cancer activity of the combined treatment were more potent than either type of nanoparticle alone or the free drugs [288].

Ongoing research into chitosan nanocomposites focuses on optimizing particle size, surface charge, and ligand density to improve targeting efficiency and drug loading capacity. Clinical trials are necessary to validate these technologies using new modified chitosan derivates, ensuring their safety and efficacy for human use. Still, the chitosan-based nanocomposites offer a promising platform for the targeted oral delivery of chemotherapeutics to treat colon cancer.

The use of chitosan and its derivatives, particularly PEGylated chitosan, has also been explored extensively for the targeted delivery of small interfering RNA (siRNA) to colon cancer cells, presenting a novel approach in gene therapy for cancer. siRNA has emerged as a promising therapeutic tool due to its ability to specifically silence disease-related genes, offering a strategic method to suppress tumor growth and proliferation. Chitosan nanoparticles are an effective carrier for siRNA due to their biocompatibility and ability to protect siRNA from enzymatic degradation in the biological environment [246, 292].

The intracellular release of small interfering RNA (siRNA) or other therapeutic payloads involves a series of physicochemical and biological mechanisms. Chitosan because of its polycationic nature is suitable for nucleic acid delivery and can be used for development of chitosan-based carriers for molecular modulation of cells. To achieve the functional activity of encapsulated siRNA several steps of chitosan-siRNA interaction with the cell are necessary starting from cellular uptake, then endosomal siRNA escape followed by cytoplasmic release and gene silencing.

1. Cellular Uptake of Chitosan-siRNA Complexes:

Chitosan forms polyplexes with negatively charged siRNA through electrostatic interactions, resulting in nanoscale particles that protect siRNA from enzymatic degradation in the extracellular environment. These complexes are typically internalized by cells via endocytic pathways, predominantly clathrin-mediated or caveolae-mediated endocytosis. The specific pathway depends on the chitosan carrier's size, surface charge, and functionalization [233].

2. Endosomal Escape

A critical step for functional gene silencing is the release of siRNA from the endosomes into the cytoplasm. Chitosan facilitates endosomal escape via the “proton sponge effect,” albeit to a lesser extent than some synthetic polymers like polyethyleneimine (PEI). The amine groups in chitosan become protonated in the acidic endosomal environment, resulting in osmotic swelling and rupture of the endosomal membrane. Additionally, chitosan derivatives, such as those modified with histidine or imidazole groups, enhance endosomal escape by improving buffering capacity and promoting membrane disruption [234].

3. Cytoplasmic release and gene silencing

Once in the cytoplasm, the siRNA must dissociate from the chitosan carrier to interact with the RNA-induced silencing complex (RISC). The efficiency of this release is influenced by the binding strength between chitosan and siRNA. Optimal design of the chitosan structure—e.g., by controlling the degree of deacetylation, molecular weight, and functional modifications—enables a balance between stability during delivery and prompt release within the cytosol [233].

4. Functional activity

Following release, the siRNA is incorporated into the RISC complex, guiding it to complementary mRNA sequences and facilitating mRNA cleavage or translational repression. This process effectively downregulates the expression of target genes, a mechanism that underpins RNA interference (RNAi)-based therapeutic strategies [37].

Recently it has been shown that chitosan particles loaded with siRNA for Cystatin C facilitate the control of intracellular drug-resistant *Mycobacterium tuberculosis* [222]. Furthermore, chitosan nanoparticles with long synthetic siRNAs have been used for targeting vascular endothelial growth factor (VEGF) in breast cancer cells [56].

However, chitosan alone has limitations regarding solubility and targeted delivery efficiency. To overcome these challenges, polyethylene glycol has been conjugated to chitosan nanoparticles. PEGylated chitosan enhances the stability of the nanoparticles in the bloodstream and improves their solubility, which is crucial for the delivery of therapeutic molecules like siRNA. The targeted delivery of siRNA using PEGylated chitosan nanoparticles focuses on enhancing the uptake by colon cancer cells while minimizing adsorption by healthy cells. This specificity is typically achieved by modifying the surface of the nanoparticles with targeting ligands that recognize and bind to receptors overexpressed on the surface of cancer cells [246, 292]. This receptor-mediated endocytosis allows the nanoparticles to deliver siRNA directly into the cancer cells, where the siRNA can exert its gene-silencing effects, effectively reducing tumor viability and progression. This approach holds significant promise for improving the precision and effectiveness of colon cancer treatment by directly targeting the genetic abnormalities that drive cancer development. Further research and clinical trials are necessary to optimize these delivery systems, evaluate their long-term safety, and establish their efficacy in patients [246, 292].

Cetuximab-conjugated chitosan-pectinate composite nanoparticles represent a sophisticated approach in targeted drug delivery systems to enhance the therapeutic efficacy against colon cancer [248]. This innovative strategy harnesses the unique properties of chitosan and pectinate alongside the tumor-targeting capabilities of cetuximab. This monoclonal antibody specifically binds to the epidermal growth factor receptor (EGFR) frequently overexpressed in colon cancer cells. Chitosan-pectinate composite nanoparticles leverage chitosan's bioadhesive properties and pectinate's gel-forming capabilities to enhance mucosal adherence and prolong gastrointestinal transit time. This prolongation is crucial for increasing the duration of contact between the therapeutic agent and the mucosal surfaces, thereby improving drug absorption and effectiveness. The encapsulation of cetuximab within these nanoparticles also protects it from enzymatic degradation within the digestive tract, ensuring its integrity until it reaches the target site. Conjugating monoclonal antibody—cetuximab to the nanoparticles is pivotal for targeting EGFR-expressing tumor cells while sparing normal cells, thereby reducing non-specific cytotoxicity and enhancing the therapeutic payload delivery directly to the tumor site. These nanoparticles are engineered to release their payload in a controlled manner, a feature that ensures sustained release of cetuximab, providing continuous engagement with cancer cells over an extended period. Additionally, the nanoparticles can facilitate the penetration of cetuximab into the tumor microenvironment, enhancing its therapeutic efficacy [248]. The development of cetuximab-conjugated chitosan-pectinate nanoparticles is a promising step forward in personalized cancer therapy, especially for patients with EGFR-positive colon cancer.

Also, chitosan and its modifications can be potentially used in gastric cancer therapy. The modification of chitosan to produce derivatives such as trimethyl chitosan (TMC) enhances its solubility and mucoadhesive properties, potentially increasing its utility in drug delivery systems [127, 253]. The focus on TMC is due to its enhanced ability to interact with the cellular membranes of cancer cells, promoting better uptake and efficacy of loaded drugs such as paclitaxel. TMC retains chitosan's biocompatible and biodegradable characteristics but exhibits increased positive charge density, enhancing its interaction with negatively charged biological membranes. Paclitaxel is a potent chemotherapeutic agent used in the treatment of various cancers, including gastric cancer. However, its clinical use is limited by its poor solubility and systemic toxicity. Encapsulating paclitaxel in TMC nanoparticles can enhance solubility, reduce peripheral toxicity, and allow controlled release. Additionally, TMC-paclitaxel nanoparticles have shown higher cytotoxicity against gastric cancer cells in vitro than free paclitaxel, suggesting that TMC facilitates better cellular uptake and retention of the drug.

As mentioned above, some proteins have antibacterial properties toward bacteria involved in the development of cancers, including *H. pylori, B. fragilis, S. enterica, F. nucleatum,* and *P. gingivalis* [108]. An example of such a protein is azurin, produced by *P. aeruginosa*. The in vitro studies proved that the immobilization of azurin on the chitosan nanoparticles significantly increased anticancer activity of azurin (as tested on the gastric cancer cell line CLS-145, pancreatic cancer cell line AsPC-1, colon cancer cell line HCT116, esophagus cancer cell line KYSE-410, and liver cancer cell line HepG2), as well as antibacterial activity against the bacteria species appearing in biopsies related to gastrointestinal cancer [11]. Öztürk et al. introduced a novel approach for repurposing the antiretroviral drug lamivudine for lung cancer treatment by loading it into nanoparticles using the nano spray drying method [212]. Chitosan nanoparticles were designed for oral delivery, enhancing drug bioavailability and targeting lung cancer cells. The study demonstrated significant improvement in drug encapsulation efficiency (above 80%) and sustained release kinetics, showing promise as effective drug carriers for repurposing antiretroviral drugs in cancer therapy.

Interestingly, chitosan, thanks to its unique properties, can readily pass through the vasculature of the liver, leading to its accumulation or even uptake by hepatocytes and its retained there. The small size of the molecules, their positive charge and additional modifications chitosan nanoparticles enhance these processes. Nanocarriers based on galactosylated chitosan, glycyrrhizin conjugated chitosan and other chitosan derivatives increased liver-targeting of drugs, prolonged its residence time as well as the concentration achieved in liver cells. Accumulation of chitosan loaded with anticancer agents in the liver, seems essential because of the key role of this organ in drug metabolism. The use of chitosan may increase the effectiveness of anticancer therapies in liver cancer while reducing their systemic toxicity. On the other hand, the use of chitosan may cause reduced drug delivery to cancer cells other than the hepatocellular carcinoma and lead to increased toxicity at the liver cell level [31, 317].

#### The management of gastroesophageal reflux disease based on chitosan's unique properties

Chitosan, due to its unique chemical and physical properties, has emerged as a promising agent in the management of gastroesophageal reflux disease (GERD), a prevalent condition characterized by the backflow of stomach acids into the esophagus, leading to symptoms such as heartburn, regurgitation, and dyspepsia. The application of chitosan in reducing gastric reflux hinges on its ability to form a gel-like barrier, its acid-binding capacity, and its bioadhesive properties, which can modify the gastroesophageal environment and potentially mitigate reflux episodes [303].

Chitosan functions primarily through its acid-neutralizing and viscosity-enhancing properties when it comes into contact with gastric contents. Upon administration, chitosan interacts with gastric acid to form a viscous solution that increases the overall gastric pH. More crucially, due to its mucoadhesive nature, chitosan can adhere to the mucosal lining of the stomach and lower esophagus. This adhesion provides a physical barrier that restricts the ascent of acidic gastric contents and reinforces the integrity of the mucosal barrier against acid-induced injury. Chitosan’s ability to form a gel barrier that floats on the stomach contents is particularly advantageous. This "raft" -like mechanism is similar to alginates used in antacid preparations, which are well-documented for their efficacy in GERD management. The raft can act as a mechanical shield for the lower oesophagus during reflux episodes, thus reducing oesophageal exposure to acid. Unlike traditional antacids, which neutralize acid through a chemical reaction, chitosan binds with gastric acid to form a non-dissolvable complex, thus diminishing the volume of free hydrochloric acid in the stomach. This action can potentially extend the duration of symptom relief compared to conventional antacids, which can suffer from rapid acid rebound. Chitosan's bioadhesive properties enable it to coat the mucosa, protecting the oesophageal tissue from erosive damage and promoting mucosal healing. This is particularly relevant in patients with erosive esophagitis, where mucosal repair is crucial for long-term management and preventing complications such as stricture formation.

Chitosan is typically administered in a capsule form designed to disintegrate in the stomach rapidly, releasing chitosan particles that can interact immediately with gastric contents. The dosage and timing of administration are critical factors to consider. Chitosan should ideally be taken postprandially to ensure that it can effectively interact with the gastric contents during potential reflux, particularly after large or fatty meals, which are known exacerbating factors for GERD. Emerging clinical trials have begun to explore the efficacy of chitosan in GERD management [128]. These studies assess symptom relief, frequency of reflux episodes, and mucosal healing as evaluated by endoscopic examinations. Early results are promising, indicating a reduction in symptom severity and frequency. However, more extensive multicenter trials are required to fully establish chitosan's role and optimize its formulation for GERD treatment.

## Conclusions

This review has been dedicated to presenting chitosan and chitosan-based nano- or microparticles and the recent biomedical application concepts on chitosan-based formulations. Chitosan, as a non-cytotoxic and biocompatible natural component, shows direct antibacterial activity and is a suitable matrix for delivering antibacterial peptides and proteins. It can be used for the development of vaccines against bacterial pathogens as well as anti-viral formulations. Health-promoting applications of probiotics or other bacterial species encapsulated in chitosan matrix have been presented. Furthermore, the potential of chitosan-based formulations to prevent the development of noninfectious diseases, including GERD or cancer diseases, has been described. The immunomodulating properties of chitosan have been found very promising in the light of different vaccination strategies and anticancer therapies. The reviewing of literature on chitosan-derived biomaterials for regenerative medicine in bone and cartilage, as well as soft tissues, delivered many examples of different efficient applications in this area. 

Chitosan is extensively used in the market, depending on the purity, in industrial, food, or pharmaceutical applications. In pharmaceutics, chitosan can serve as a diluent for tablets, a disintegrant, a matrix for DDS, wound dressings or an absorption enhancer. The chitosan-based wound dressings are predominantly available on the market in the form of non-wovens, films or sponges. The FDA-approved products are as follows: Hemcon ^®^bandage, Guardacare ® XR surgical, Chito Flex ^®^ PRO, Chito gauze^®^ XR PRO, Celox ^™^ Gauze, Celox Gauze, AxioStat, MaxioCel [221]. There are also dietary supplements: Inlife Chitosan Supplement, NOW Foods Chitosan, Prozis Chitosan + Vitamin C, OLIMP Chitosan Chrom, Naturhouse Chitosan, etc. However, the translation of nanomedicines from the lab scale into clinical trials is ongoing with limited success. According to the database (ClinicalTrials.gov), various chitosan nanomedicines are under examination: NCT03588351 (root canal bacteria), NCT06567301 (knee osteoarthritis), NCT06533215 (*Enterococcus faecalis* infection), NCT06782087 (tooth whitening and color stability), NCT06523244 (photodynamic therapy), NCT06926322 (non-vital primary molars). Those trials show a great potential in chitosan-based DDS; however, due to poor reproducibility and batch-to-batch variations of chitosan, this goal still needs to be reached. Nevertheless, the ongoing clinical trials prove that those obstacles can be overcome to improve manufacturing time, reduce batch-to-batch irreproducibility, and production on an industrial scale-up could soon be possible. Efforts should be made to follow the International Conference on Harmonisation (ICH) guidelines of Technical Requirements for Pharmaceuticals for Human Use to achieve the requirements for pharmaceutical product registration. 

Concerning the future research directions, to optimize of chitosan functionalization to improve targeted drug delivery is of great importance. It is possible to modify chitosan to strengthen its interactions with target cells and tissues, enhance drug loading and release, and diminish off-target effects. Such modifications can include hydrophobic alterations, ligand attachments, and pH-responsive methods. Furthermore, chitosan can be combined with other components or drugs to obtain synergistic effects, boosting drug efficacy while reducing side effects [214, 219]. Incorporating hydrophobic components amplifies chitosan's capacity to interact with cell membranes, thereby facilitating cellular uptake and improving drug delivery. Recent research suggests that hydrophobic modifications can enhance the cellular uptake and therapeutic efficacy of chitosan-based drug delivery systems [224]. Quaternization increases the positive charge of chitosan, thereby enhancing its interaction with negatively charged cell surfaces. Thiolation improves mucoadhesion, which facilitates drug delivery in the gastrointestinal tract. The incorporation of polyethylene glycol (PEG) chains enhances the stability of chitosan and reduces its immunogenicity. Improving the mucoadhesive properties and biological interactions of chitosan can be accomplished by functionalizing amino groups, while hydroxyl groups considerably enhance chitosan's solubility and stability [289]. Functionalizing chitosan with ligands such as antibodies or peptides facilitates precise targeting of cancer cells and other specific tissues. For instance, chitosan nanoparticles with ligand modifications have been developed for the targeted delivery of drugs to cancer cells, demonstrating enhanced accumulation in tumors and greater efficacy [214]. Modifying chitosan by incorporating pH-sensitive groups facilitates customized drug release at specific pH, particularly in the acidic milieu of tumor cells. Chitosan-based micro/nanocapsules with pH-responsive characteristics are currently being developed for effective drug delivery in the acidic tumor microenvironment [219].

## Data Availability

Not applicable.

## Abbreviations

AC: Alginate/chitosan
ACAC: Double bilayer ones
AMPs: Antimicrobial peptides
APC: Antigen presenting cells
APEC: Avian pathogenic *E. coli*
AsPC-1: Pancreatic cancer cell line
BabA: Blood antigen binding adhesion
BAX: Bcl (B-cell lymphoma 2),-2-like protein 4
BCG: Bacillus Calmette-Guerin
bFGF: Basic fibroblast growth factor
BMPs: Bone morphogenetic proteins
CHAP: Chitosan/polyethylene oxide
CMCS: Carboxymethyl chitosan
CRC: Colorectal cancer
CTA: Cinnamaldehyde-based thioacetal
CTB: Cholera toxin B subunit
DD: Deacetylation degree
DDSs: Drugs delivery systems
De-HACC: Hydroxypropyltrimethyl ammonium chloride fully deacetylated chitosan
EBs: Elementary bodies
EGFR: Epidermal growth factor receptor
EHEC: Enterohemorrhagic *E. coli*
EspA: *E. coli* secretion protein A
EV71: Vaccine formulation toward enterovirus 71
FDA: Food and Drug Administration
GERD: Gastroesophageal reflux disease
GM-CSF: Granulocyte–macrophage colony stimulating factor
GOT: Glutamic oxaloacetic transaminase
GPT: Glutamic pyruvic transaminase
GRAS: Generally regarded as safe
GRAS: Generally regarded as safe
GTMAC: Glycidyl trimethyl-ammonium chloride
HACC: Hydroxypropyltrimethyl ammonium chloride chitosan
HCT116: Colon cancer cell line
HepG2: Liver cancer cell line
HIV: Human Immunodeficiency Virus
Hsp: Heat shock protein
IDR: Innate defense regulator
IFN- γ: Interferon gamma
Ig: Immunoglobulin
IL: Interleukin
KYSE-410: Esophagus cancer cell line
LbL: Layer-by-layer
LDH: Lactate dehydrogenase
Leb: Lewis b
LT: Labile-toxin
LTAs: Lipoteichoic acids
MCP-1: Macrophage chemotactic protein -1
MCs: Microcontainers
MIC_50_: Half of minimal inhibitory concentration
MRSA: Methicillin-resistant *Staphylococcus aureus*
MSC: Mesenchymal stem cells
MTT: (3-(4,5-Dimethylthiazol-2-yl)-2,5-diphenyltetrazolium bromide
NBT: Nitro blue tetrazolium
NIS: Nisin
NK: Natural killer cell
NLRP3: NLR—Nod-like receptor family pyrin domain containing 3
NO: Nitrogen oxide
OLA: Oleylamine-based zwitterionic lipid
OMPs: Outer membrane proteins
PEO: Cysteine/histidine-dependent amidohydrolase/peptidase
PsaA: Pneumococcal surface protein A
RANTES: Regulated on Activation, Normal T cell Expressed and Secreted chemokine
rhBMP-2: Recombinant human bone morphogenetic protein-2
rHBsAg: Recombinant hepatitis B surface antigen
ROS: Reactive oxygen species
SabA: Sialic acid binding adhesion
siRNA: Interfering RNA
SseB: Salmonella pathogenicity island 2 type III secretion system
STING: Stimulator of interferon genes
Tas: Teichoic acids
Th: T helper lymphocytes
Tir: Translocated intimin receptor
TLR: Toll-like receptor
TMC: Trimethyl chitosan
TNF: Tumor necrosis factor
TPP: Tripolyphospahate
TPP: Sodium tripolyphosphate
VCG: Vibrio cholerae ghost
VEGF: Vascular endothelial growth factor
VM: Vancomycin
VM-OLA-LPHVs1: Chitosan-based pH-responsive lipid-polymer hybrid nanovesicles
WHO: World Health Organization
